# Combining Computational Screening and Machine Learning to Predict Metal–Organic Framework Adsorbents and Membranes for Removing CH_4_ or H_2_ from Air

**DOI:** 10.3390/membranes12090830

**Published:** 2022-08-25

**Authors:** Huilin Li, Cuimiao Wang, Yue Zeng, Dong Li, Yaling Yan, Xin Zhu, Zhiwei Qiao

**Affiliations:** 1 Guangzhou Key Laboratory for New Energy and Green Catalysis, School of Chemistry and Chemical Engineering, Guangzhou University, Guangzhou 510006, China; 2 Joint Institute of Guangzhou University & Institute of Corrosion Science and Technology, Guangzhou University, Guangzhou 510006, China

**Keywords:** metal–organic framework, membrane, molecular simulation, computational screening, machine learning

## Abstract

Separating and capturing small amounts of CH_4_ or H_2_ from a mixture of gases, such as coal mine spent air, at a large scale remains a great challenge. We used large-scale computational screening and machine learning (ML) to simulate and explore the adsorption, diffusion, and permeation properties of 6013 computation-ready experimental metal–organic framework (MOF) adsorbents and MOF membranes (MOFMs) for capturing clean energy gases (CH_4_ and H_2_) in air. First, we modeled the relationships between the adsorption and the MOF membrane performance indicators and their characteristic descriptors. Among three ML algorithms, the random forest was found to have the best prediction efficiency for two systems (CH_4_/(O_2_ + N_2_) and H_2_/(O_2_ + N_2_)). Then, the algorithm was further applied to quantitatively analyze the relative importance values of seven MOF descriptors for five performance metrics of the two systems. Furthermore, the 20 best MOFs were also selected. Finally, the commonalities between the high-performance MOFs were analyzed, leading to three types of material design principles: tuned topology, alternative metal nodes, and organic linkers. As a result, this study provides microscopic insights into the capture of trace amounts of CH_4_ or H_2_ from air for applications involving coal mine spent air and hydrogen leakage.

## 1. Introduction

With the development and advancement of society and the economy, the demand for fossil energy fuels is increasing with each passing day, leading to a widespread problem of CH_4_ leakage in fossil energy extraction, production, transportation, and application. For instance, leaking in underground boreholes for gas extraction can diminish CH_4_ production and contaminate groundwater, and CH_4_ also volatilizes in gas stations. More noteworthy is that the total amount of coal mine methane (coal mine gas with methane concentration below 0.75%) is huge. During the mining process, the total amount of methane emitted is about 28 billion m^3^/a, which is equivalent to 420 million tons of CO_2_, accounting for 8% of the total anthropogenic methane emissions. Moreover, combined with its low viscosity and the technical difficulty of its use, this methane has had to be exhausted entirely into the atmosphere for long periods, causing significant greenhouse gas pollution. Apart from fossil fuels, natural gas, hydrogen energy, and hydrogen fuel cells are also developing rapidly owing to their high green energy value. However, one of the main factors hindering the commercialization of hydrogen-fueled vehicles is the safety and cost caused by the leakage of H_2_. Meanwhile, CH_4_ and H_2_ are clean energy carriers with high calorific value and low carbon emissions. It would be progress if we could capture this part of the H_2_ and CH_4_ free in the air. Therefore, determining how to separate and capture the relatively small amounts of CH_4_ and H_2_ from air (O_2_ and N_2_) has become the focus of research. Exploiting diverse new adsorbents or separation membranes is the key for the safe and cost-effective storage and separation of gases (CH_4_ and H_2_).

In recent years, a newly developed category of porous coordination polymer materials has attracted significant research attention and received widespread attention in scientific research and industry. Obtained by self-assembly of metal ions and organic ligands, metal–organic frameworks (MOFs) [1,2,3] not only have crystalline structures similar to the regular pores of zeolite molecular sieves but also have higher specific surface areas than conventional porous materials, and organic components make them both designable and tailorable, with adjustable pore sizes and easy functionalization of the channel surfaces. Based on these structural features, which act as adsorbents, MOF materials can achieve high-density energy storage of clean fuel gases. MOFs can also generate differential interactions for different gas molecules to achieve economical and energy-efficient separation of gases, and they are therefore widely used in gas adsorption and separation [4,5,6,7], storage [8], catalysis [9,10], drug transport [11], and sensing applications [12], especially in the field of gas adsorption and separation in which a number of breakthroughs have been accomplished. Chang [13] showed in breakthrough experiments that a microporous MOF (SBMOF-1) allowed good separation of methane from N_2_. Xu [14] found it effective to combine B-substitution and Li-decoration or C_48_B_12_Li insertion as a strategy to improve the capacity of CO_2_ to absorb H_2_ and methane and to separate it from CO_2_/CH_4_ and CO_2_/H_2_ mixtures. This multiple modification strategy opened a door for the production of porous nanomaterials with higher gas adsorption and separation capabilities. Kang [15] found in his study that MOFJUC-150 membranes have a significant preferential permeability to H_2_ compared to other gas molecules. At ambient temperature, the selectivity factors of the membranes for H_2_/CH_4_, H_2_/N_2_, and H_2_/CO_2_ were 26.3, 17.1, and 38.7. MOFs have also been reported to possess ultra-high surface areas, especially for a series of CH_4_/H_2_ separations, with higher selective adsorption capacities than conventional porous materials, indicating that such materials are the most attractive adsorbent materials for CH_4_/H_2_ separation [16,17]. Eddaoudi and co-workers [18] developed several novel MOF materials for efficient gas storage, and one of them, a hybrid material, could carry out the cost-effective storage of methane.

MOFs have experienced rapid development over the past decade. In addition to being used as adsorbents, the properties of MOF membrane materials make them suitable for gas separation. Kang et al. [19] summarized the advances in improving the performance of MOF membranes (MOFMs) in recent years, including practical application problems faced by the design and growth of MOFs. Separation processes by using membrane materials have shown to be efficient, have low energy costs, can be easily performed [20], and have advantages in terms of environmental safety and scalability. Conventional materials, such as carbon, zeolites, and polymers, have been explored as membranes for the separation of natural gas. However, the number of zeolite structures and the diversity of zeolite membranes are restricted, and it is difficult to control the precise pore size and pore function of zeolites [21]. Polymeric membranes, however, are often subject to a balance between the permeability and selectivity (called Robson’s upper limit); that is, the increase in the permeability in conventional membrane materials occurs at the cost of selectivity [22]. Nevertheless, MOF membrane materials show strong potential. Hou et al. [16] reported a 10-fold higher separation coefficient, up to 25, for ZIF-722-8 membranes in CO_2_/methane mixtures. Liu’s group [23] found that fcu-MOF membranes exhibited good permeability and selectivity for the separation of H_2_/CO_2_/N_2_, CO_2_/CH_4_, and N_2_/CH_4_ mixtures. Some MOF membrane materials even exceeded Robson’s upper limit. For example, Wang [24] developed the ZIF-62MOF glass membrane with separation coefficients of 50.7, 34.5, and 36.6 for H_2_/CH_4_, CO_2_/N_2_, and CO_2_/CH_4_ mixtures, respectively.

To date, although a large number of MOFs have been synthesized and reported, the number is almost infinite due to the large number of possible metal ions and organic linkers. Consequently, it is inefficient to filter MOFs from a large database for a specific application for which the high-throughput computational screening (HTCS) method based on molecular simulations provides a suitable alternative. Numerous studies [25,26,27,28] have shown that the method of discovering high-performance target materials and mining the quantitative structure–property relationships (QSPRs) from the large number of MOFs provide is effective for selecting superior MOFs on a large scale. However, many inefficient computations are performed during high-throughput computing, resulting in a considerable waste of computational resources and valuable research time. Recently, the emergence of machine learning (ML) has made up for this shortcoming. ML has been gradually applied in many fields, such as material discovery, structure analysis, property prediction, and reverse design, and has shown high potential in materials research [6,29,30,31,32]. Li et al. [33] summarized the latest advances in the use of MOFs for gas storage from the three aspects of H_2_, CH_4,_ and C_2_H_2_. In addition to gas storage, it highlights some of the significant advances made by MOF materials in the separation of important gases in recent years. According to Shi et al.’s work, ML models were trained to be two to three orders of magnitude faster than HTCS. The combination of ML and simulation techniques is an effective way to find the optimal MOF quickly and with the greatest probability [6]. For example, Yan et al. [34] screened the dynamic adsorption of O_2_ and N_2_ in 6013 computation-ready experimental MOFs (CoRE-MOFs) by using ML and large-scale calculations. They also systematically analyzed the influences of all the metals in the periodic table on the performances of the materials and proposed six rational design criteria for adsorbent materials, successfully designing a series of materials with superior performances. Recently, Jiang and co-workers [35] deployed a hierarchical approach using molecular simulations and machine learning to rapidly screen 100,000+ MOFs that were synthesized experimentally for C_3_ separation, and they used trained ML models for rapid screening of other MOF datasets (e.g., experimental Cambridge Structural Database (CSD) MOFs and hypothetical MOFs). For the CSD MOFs, the out-of-sample predictions closely coincided with the simulation results, which indicated good transferability of the ML model from the CoRE-MOFs to CSD MOFs. Furthermore, nine CSD MOFs that exhibited better separation performances than the best performing CoRE-MOFs were revealed. Rosen et al. [36] trained an ML model on a quantum database (QMOF) with 14,000 MOFs to discover MOFs with structural properties of the target electrons in a rapid manner. Through reconstruction of the typical track of failed experiments, Moosavi et al. [37] reported an ML approach to obtaining chemical intuition and successfully searched for the optimal synthesis conditions, which yielded the highest surface area of HKUST-1 reported to date. He also illustrated the importance of quantifying this intuition for the synthesis of new materials. Azar [38] predicted the H_2_ permeability and H_2_/N_2_ selectivity from 3765 different types of MOFMs, and the results demonstrated that MOFMs had a high H_2_ permeability, 2.5 × 10^3^ to 1.7 × 10^6^ barrer. Qiao et al. [26] identified five optimized MOFs at 298 K and 10 bar by simulating the adsorption, diffusion, and permeation of CO_2_/N_2_/CH_4_ mixtures in 24 samples using a computational study of the high-throughput screening of 137,953 MOFs. Qiao also selected 4764 CoRE-MOFs for membrane separation of ternary gas mixtures (CO_2_/N_2_/CH_4_) at 298 K and 10 bar and finally identified seven PLDs of the best MOFs with 2.91–3.26 Å and pore size distribution (PSD% _(2.4 to 3.5 Å)_) of 48.2–64.1% [39]. Recently, Bai et al. [40] targeted computational screening 6013 MOFMs for H_2_ separation and explored the relationship between material characteristics and properties from the perspective of ML. The main difference with Bai’s research is the separation of trace amounts of CH_4_ (or H_2_) and ternary systems in this work. All these again confirmed that the synergistic use of ML and HTCS is an effective way to achieve faster, better predictions and probability maximization of high-performance MOFs or MOFMs.

Gas separation using MOFs is generally divided into two categories: equilibrium-based gas separations and kinetic-based gas separations [41]. In equilibrium-based gas separations where MOFs are used as adsorbents, its selectivity of equilibrium-based separations was controlled by the affinity of MOFs for adsorption of one gas relative to another. In kinetic-based separations, the selectivity is controlled by the combination of adsorption and diffusion, being determined by the different transport rates of the gas species through the membrane pores, and MOFs are used as membranes [42]. Admittedly, the use of membrane separation for processing trace gases requires a larger driving force and increases cost; however, MOFMs have certain properties and their applicability to specific sites that are superior to MOF adsorbents and other types of membrane materials. Firstly, the present simulations were performed under atmospheric pressure and temperature conditions, which are applicable to general situations. Secondly, MOF adsorbents are less stable in aqueous or high heat environments, while membranes have stronger stability, e.g., ZIF-90 membranes show good steadiness in the presence of steam [43]. Therefore, membrane materials may be more advantageous when operating in liquid or high temperature environments. Gokay Avci et al. [44] also proposed that the separation of H_2_ is more advantageous in membrane applications than CO_2_ because H_2_ has a higher molar fraction and a smaller kinetic diameter, which allows H_2_ to penetrate smaller membrane pores more quickly. In addition, MOFMs are better equipped for further modification and refinement of the material, e.g., as polymer-filled particles in polymers to improve polymer separation performance, when making MOF-based MMM is much more economical than making pure MOF membranes, since the former requires only a small amount of MOF [43]. MOFs have good potential in pressure-driven membrane processes, as well as in selective filtration or permeation-driven membrane processes. After comprehensive consideration, in some specific occasions, MOF membranes will have better performance and more economical applications. In summary, it is also necessary to develop materials with good membrane separation performance.

The purpose of this work was to investigate the adsorption, diffusion, and permeation properties of 6013 MOF adsorbents and MOFMs for the capture of clean energy gases (CH_4_ and H_2_) in air using large-scale computational screening and ML. In Section 2, we described atomic models of CoRE-MOFs/MOFMs and gases, simulation methodologies, and machine learning principles. In Section 3, we discussed the relationships between the adsorbent performance metrics and their characteristic descriptors. With the help of ML techniques, we found the best ML model for a particular performance metric aimed at CH_4_/O_2_ + N_2_ and H_2_/O_2_ + N_2_ mixtures. Furthermore, this algorithm was applied to quantify the relative importance of the performance metrics for each MOF descriptor. Finally, high-performance MOF materials for different systems were identified, and commonalities between the high-performance MOFs were evaluated to propose design principles.

## 2. Models and Methods

### 2.1. Model

In this work, we used molecular simulation to screen 6013 computation-ready, experimental MOFs (CORE-MOFs) for their ability to capture energy gases (CH_4_ and H_2_) from air. All crystal structures were taken from the Cambridge Crystallographic Data Centre (CCDC), whose parameters were compiled and validated by Chung et al. [45]. Each MOF is described by five structural descriptors (largest cavity diameter (LCD, Å), void fraction (*ϕ*), volumetric surface area (VSA, m^2^/cm^3^), pore limiting diameter (PLD, Å), density (*ρ*, kg/m^3^)) and two energy descriptors (heat of adsorption (*Q*_st_, kJ/mol) and Henry’s constant (*K*, mol/kg/Pa)). Specifically, N_2_ with a diameter of 3.64 (Å) and He with a diameter of 2.58 (Å) were used as probes in RASPA to calculate VSA and *ϕ*. Then, LCD and PLD were calculated using the Zeo++ [46] package. *K* was simulated in RASPA using the NTV-MC Scheme (N is the number of molecules, T is the temperature, V is the volume, and MC denotes Monte Carlo). The structural atoms of MOFs are described by the Lennard–Jones (LJ) potential and electrostatic potential, and all the LJ potential parameters are derived from the universal force field (UFF) [47], which is listed in Table S1. The atomic charges of MOFs can be quickly calculated using the MOF electrostatic-potential-optimized charge equilibration (MEPO-Qeq) method, which helps to accurately screen MOFs to adsorb specific gases. The force field parameters of CH_4_, H_2_, O_2,_ and N_2_ are derived from the TraPPE force field, which is listed in Table S2. A number of studies have verified that the UFF force field, the MEPO-Qeq charge algorithm, and the TraPPE [48] force field can accurately predict the adsorption and diffusion of gases in different MOFs materials [49,50]. 

### 2.2. Simulation Method

We simulated the adsorption and diffusion characteristics of 6013 CoRE-MOFs for airborne capture of energy gases (CH_4_ and H_2_) using grand canonical MC (GCMC) and Molecular Dynamics (MD) at 1 bar and 298 K. The interactions between MOFs and adsorbent molecules were calculated by the Lorentz–Berthelot rule, and periodic boundaries were imposed in the three-dimensional system to simulate the cell expansion along the three-dimensional direction to at least 24 Å. To calculate the LJ interactions, the spherical truncation radius for the long-range correction was set to 12 Å. The electrostatic interactions between the frame and gas molecules and between the gas molecules were calculated using the summation of Ewald [51]. In each MOF, the MC simulation was run for 100,000 cycles. The first 50,000 cycles were used to equilibrate the simulated system, and the second 50,000 cycles were used to calculate the average value of the pendulum amplitude. Each cycle consisted of tests for experiment with n (n: number of adsorbed molecules including translation, rotation, regeneration, and exchange). One GCMC [52] and MD simulation were run independently for each of the 6013 MOFs. In the MD simulations, for each ternary gas mixture system in each MOF the MD duration was 7 ns, and the last 5 ns was used for production. It was found that further increases in the number of cycles and MD duration had little effect on the simulation results. All simulations were run under the RASPA package.

### 2.3. Machine Learning

The rapidly evolving field of ML-assisted materials research and development has made it possible to combine materials databases and machine learning methods to drive materials discovery and design and predict material properties. The machine learning part of this work was performed using a Python-based automated machine learning development tool (tree-based pipeline optimization tool (TPOT)), a fast model selection and tuning method based on genetic algorithms. In addition, two machine learning algorithms (decision tree (DT) and random forest (RF)) from the Scikit-learn package [53] in Python 3.9 were used for each performance metric (adsorption selectivity (*S*_ads_), diffusion selectivity (*S*_diff_), diffusion coefficient (*D*), permeability (*P*), and permselectivity (*S*_perm_)) for each sorbent (CH_4_ and H_2_). It is worth mentioning that the first three performance indicators correspond to MOF adsorbent applications, and the last two performance indicators correspond to MOF membrane applications. More details of the algorithm are shown in Figures S6–S8. For each ML method, the data set was randomly divided into a training set and a test set, where 80% was used for training the model and the rest for testing. We used k-fold cross-validation (*k* = 5) for model construction to reduce the effect of data partitioning during learning, and we set a fixed random seed to ensure the reproducibility of the results. Each algorithm was repeated five times in our work, and the final results were averaged for each performance metric prediction. In addition, the accuracy of the models was evaluated using the Pearson correlation coefficient (*R*), mean absolute error (MAE), and root mean square error (RMSE). More details can be found in the Supporting Materials (SM).

## 3. Results and Discussion

In this work, 6013 CoRE-MOFs were used to adsorb and separate mixtures of energy gases (CH_4_ or H_2_ at 1000 ppm) with air (N_2_:O_2_ = 78:21), an initial concentration that mimics the CH_4_ concentration of spent coal mine air and the concentration of a small amount of H_2_ leaking inside a confined hydrogen fuel cell. This separation aim is the capture of CH_4_ or H_2._ With this micro level at atmospheric pressure (1 bar), it can also be theoretically shown that it makes sense to the separation of CH_4_ or H_2_ in air. In order to design superior adsorbents or membranes, first, the relationships between seven structural/energetic descriptors (LCD, *ϕ*, VSA, PLD, *ρ*, *Q*_st_, and *K*) of 6013 CoRE-MOFs and the static adsorption, diffusion performance, and permeation properties (*D*, *S*_ads_, *S*_diff_, *P*, and *S*_perm_) were explored by univariate analysis for two systems (CH_4_/N_2_ + O_2_ and H_2_/N_2_ + O_2_). The relationships were obtained, and the preliminary influence characteristics affecting the adsorption and diffusion of CH_4_ (or H_2_) in air were obtained. Then, the complex structure–property relationships were further explored by machine learning methods (TPOT, DT, and RF). Finally, the best candidates were identified for different applications, and the corresponding design principles of adsorbent/membrane materials were proposed.

### 3.1. Adsorption and Diffusion

Understanding the correlation between the diffusion coefficients of CH_4_ (or H_2_) for N_2_ and O_2_, adsorption selectivity, and diffusion selectivity as well as the structure/energy descriptors of the MOFs in terms of both adsorption and diffusion separation properties can help to uncover materials with potential for specific applications. Figures S1 and S2 show highly similar trends in the structure–property relationships in both systems, so only the CH_4_/N_2_ + O_2_ hybrid system was elaborated. Figures S1a–d,g clearly show that *D*, *S*_ads_, and *S*_diff_ first increased and finally leveled off as the five descriptors (LCD, *ϕ*, VSA, PLD, and *K*) increased. When the pore size was small, the pore walls of different framework materials exhibited strong and weak adsorption of gases, and high variability in the selectivity occurred. As the pore size increased, both N_2_ and O_2_ entered the MOFs in large amounts, and the selectivity decreased dramatically and eventually converged to one for both the CH_4_/N_2_ + O_2_ and H_2_/N_2_ + O_2_ systems. The *ϕ*, VSA, and PLD showed similar relationships, which was consistent with the previously reported trends [25,54]. It is worth noting that in Figure S1e, three performance metrics, *S*_ads_, *S*_diff_, and *D*, all tended to decrease as *ρ* increased. This was because a higher density of an MOF corresponded to a very dense internal space. As shown in Figure S1f, there were no significant trends of the three performance indicators, *S*_ads_, *S*_diff_, and *D*, with *Q*_st_, which indicated the insignificant role of *Q*_st_.

**Figure 1:**
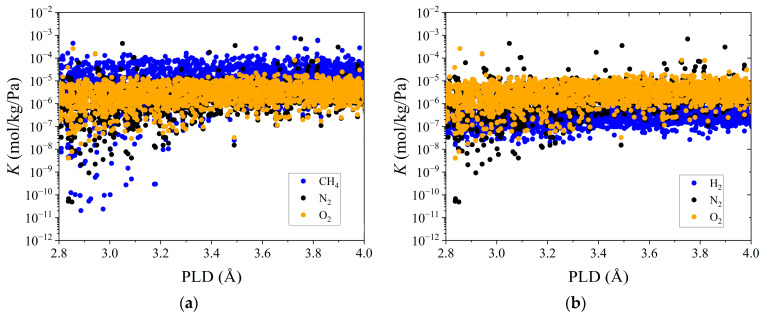
(**a**) Henry’s constant *K* vs. PLD for CH_4_, N_2_, and O_2_ in 6013 CoRE-MOFs; (**b**) Henry’s constant *K* vs. PLD for H_2_, N_2_, and O_2_ in 6013 CoRE-MOFs.

**Figure 2:**
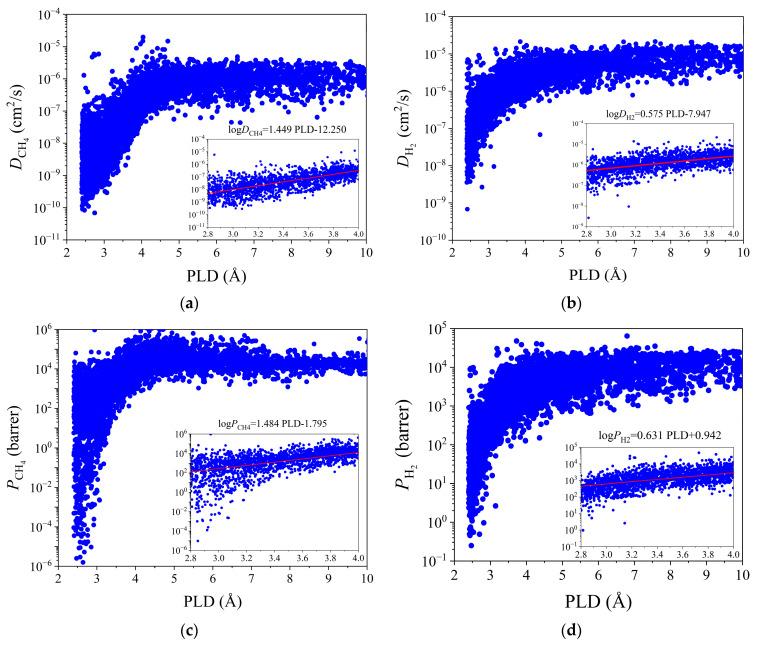
Diffusion coefficient *D* and permeability *P* vs. PLD for CH_4_ and H_2_ in 6013 CoRE-MOFs: (**a**) *D*_CH4_–PLD; (**b**) *D*_H2_–PLD; (**c**) *P*_CH4_–PLD; (**d**) *P*_H2_–PLD.

In addition to adsorption and diffusion selectivity, Henry’s constant of CH_4_ (or H_2_) reflects the adsorption performance of CH_4_ (or H_2_) in an infinitely dilute state and helps to explain the trapping performances of the MOFs in air with very low CH_4_ (or H_2_) concentrations. Figure 1 clearly shows the trend of the K values with PLD for the four gases. The trends of *K* for N_2_ and O_2_ were almost the same. In general, *K* decreased with CH_4_ > N_2_ (≈O_2_) > H_2_ because CH_4_ had the highest affinity for most MOFs, while H_2_ had the weakest affinity. This is the reason that CH_4_ and H_2_ could be separated in CH_4_/O_2_/N_2_ and H_2_/O_2_/N_2_ mixtures, respectively. Then, the quantitative relationship log *D* = aPLD − b was established based on the diffusion coefficients of CH_4_, H_2_, N_2_, and O_2_ at an infinite dilution and with PLD, as shown in Figure 2a,b. For most of the MOFs, *D*_H2_ > *D*_O2_ > *D*_N2_ > *D*_CH4_, and according to Table S3, this was because the gas molecules with smaller kinetic diameter diffused more quickly than their larger counterparts. This conforms to the conclusions reached in previous reports [26,34]. The above univariate analysis could only tentatively determine the relationship between the individual parameters and performance. We further utilized the ML algorithms to systematically and comprehensively analyze the integrated structure–performance relationship for the MOFs.

### 3.2. Permeation

To explore the separation performances of MOF membranes, two commonly used membrane separation performance metrics were further calculated: the permeability *P* (*P* = *K* × *D*) and the permeation selectivity *S*_perm_ (*S*_perm_ = *S*_ads_ × *S*_diff_). An evaluation method [26,40,55] has been shown to be applicable for calculating MOF membrane performances in multiple studies. Figure 2c,d show the *P* versus PLD plots of CH_4_, H_2_, N_2_, and O_2_ for 6013 MOFs. As shown in Figure 2a,b, the variation of *P* with PLD was essentially similar to that of *D* with PLD because *P* = *KD,* and *K* was almost independent of PLD. Therefore, there was also a quantitative relationship between log *P* and PLD: log *P* = cPLD – d. Slopes c for CH_4_, N_2_, O_2_, and H_2_ were 1.484, 1.134, 0.827, and 0.631, respectively. It is clear that the magnitude of the slope was positively correlated with the molecular dynamic diameters of the four gases. The permeability of CH_4_ was most affected by PLD, and the permeability of H_2_ was least affected by PLD because CH_4_ had the largest molecular diameter of the four gases, while H_2_ had the smallest. The effects of other parameters, LCD, *ϕ*, VSA, and PLD, on *P* and *S*_perm_ are shown in Figures S3 and S4. For the CH_4_/O_2_ + N_2_ system, the trends of the structure–property relationships were almost the same as those in Figures S1 and S2. In contrast, for H_2_/O_2_ + N_2_, Figure S4 shows similar variations of *P* with the seven structural parameters to those of the CH_4_ system. However, *S*_perm_ showed a completely opposite trend with the structural parameters LCD, *ϕ*, VSA, PLD, and *K*. As shown in Table S3, this was mainly because O_2_ and N_2_ had higher molecular dynamics diameters than H_2_ and smaller kinetic diameter than CH_4_. Small-pore MOFMs had different permeabilities to different size gas molecules, and this difference in permeability eventually led to a trend opposite to that of the permeation selectivity. *S*_perm_ decreased with an increase in any of the above five MOF descriptors. When PLD was small, the H_2_ molecules could pass through the MOFMs, whereas the N_2_ and O_2_ molecules could hardly pass through. Thus, it possessed a high *S*_perm_. When PLD increased slowly, the other two gas molecules could pass through the MOFMs freely; thus, *S*_perm_ decreased significantly and eventually tended to one. *S*_perm_ had a weakly decreasing trend with increasing *ρ*, as shown in Figure S4e. This was because high-density MOFMs had only small free spaces that were permeable to gas molecules, which led to low permeability values. However, some of the discrete points exhibited high *P*_H2_ values at >2000 kg/m^3^. These MOFs had relatively large free spaces, and they were composed of very heavy metal atoms (e.g., gold, platinum, and uranium), consistent with the findings of previous studies [39,40]. A variation trend of *S*_perm_ with *Q*_H2_ is not evident in Figure S4f, and most of the MOFMs exhibited moderate *Q*_H2_ values (8–12 kJ/mol). The greater the gas adsorption heat of the material was, the stronger the forces between the H_2_ gas molecules and the surface of the adsorbent material were, and the better the selectivity was. However, at the same time, energy cost required for desorption was higher, so a good adsorbent material should have a suitable adsorption heat. From the above analysis, it can be seen that it is not possible to obtain MOFMs with excellent performances based on a single descriptor. To understand the complex relationships between multiple descriptors and each performance metric, ML was further used to analyze the constitutive relationships of the MOFMs.

### 3.3. Machine Learning

Through univariate analysis, the relationships between the MOF structure/energy descriptors and adsorption/membrane separation performance were preliminarily obtained. In order to further understand the deeper relationships between the material performance and structure, especially the comprehensive effects of various MOFs descriptors on the performance and the ranking of the influence effects, we introduce an automatic machine learning development tool, TPOT. TPOT uses the structural characteristics and target performances of the MOFs as inputs and automatically generates models to achieve regression predictions of the target performances. At the same time, the best code pipeline for different target performances can be derived for further optimization and learning. In addition, two commonly used ML algorithms were employed: DT and RF. DT is a very commonly used ML algorithm. It achieves regression predictions by building a binary tree. The decision tree model is easy to implement and has strong interpretability. The RF algorithm is composed of multiple decision trees, which improves the generalization and fault tolerance of DT. 

**Figure 3:**
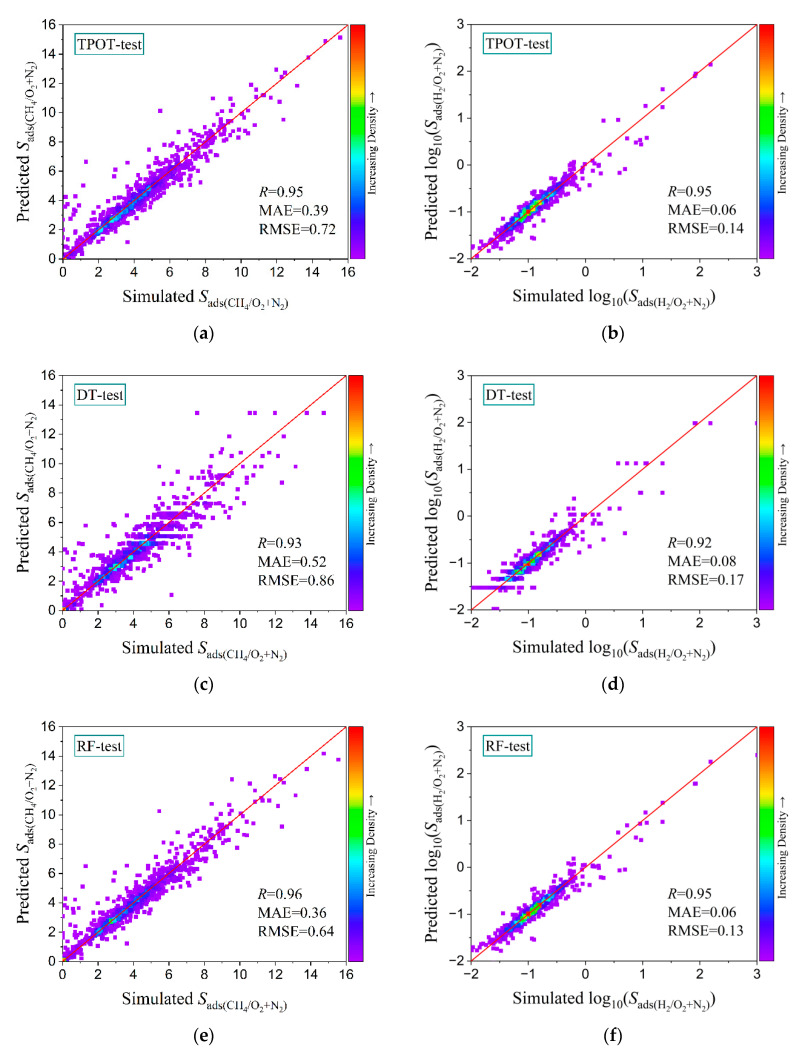
*S*_ads(CH4/O2+N2)_ and log_10_(*S*_ads(H2/O2+N2)_) predicted by three ML methods: (**a**,**b**) TPOT; (**c**,**d**) DT; and (**e**,**f**) RF versus the simulated values on the test set.

In order to find a machine learning method suitable for this system and obtain better prediction results, we used the TPOT, DT, and RF algorithms to predict each performance index (*D*, *S*_ads_, *S*_diff_, *P*, and *S*_perm_). All the results are shown in Table 1 and Figure 3. It can be seen from Table 1 that the three algorithms yielded good predictions (*R* ≥ 0.85) for all the performances of the two systems, except that the prediction effect of H_2_ on the diffusion selectivity of N_2_ and O_2_ was not good. The TPOT and RF models had good prediction abilities for the prediction of the adsorption selectivity *S*_ads_ and permeability *P*, and their *R* values were above 0.97 (see Figure 3). It is worth noting that, to narrow down a poor fitting effect caused by data span, we used logarithms to analyze *S*_ads(H2/O2+N2)_, *S*_diff(H2/O2+N2)_, *P*_(CH4/O2+N2)_, and *S*_perm(CH4/O2+N2)_. It can be seen from Figure 3 that the predictions of the RF model for the CH_4_ adsorption selectivity and gas permeability were the closest to the simulation values and had the best prediction effect. The *R* values of *S*_ads(CH4/O2+N2)_ and *P*_(CH4/O2+N2)_ on the test set reached 0.96 and 0.99, the MAE values were 0.36 and 0.26, and the RMSE values were 0.64 and 0.36, respectively. In terms of adsorption and separation, the prediction effects of the three ML models on the performance indicators of the two systems were in the order of *S*_ads_ > *S*_diff_ > *D*. However, in terms of membrane performance, for the three ML models, although the *R* values of *S*_perm_ and *P* in the test set were similar, the MAE and RMSE of *P* were more than 1000 times those of *S*_perm_, which was caused by the characteristics of the data.

In conclusion, for the prediction of all the performance indicators, the RF prediction effect was generally better than that of the DT model for different gas mixture systems, which may have been because the RF model has a strong generalization ability. In terms of the adsorption performances of the two systems, the TPOT algorithm was better than the DT algorithm for most of the predictions. This may have been because TPOT could find the best combination of models and parameters by using a genetic algorithm, which led to better prediction results on different data sets. Although the prediction results of RF and TPOT were similar, the *R* and error (MAE and RMSE) values of most of the performance indicators showed that the prediction accuracy of the RF algorithm was slightly higher than that of TPOT. Therefore, RF was considered to be an optimal, stable, and useful algorithm for the MOF system in this work. In many previous studies, the RF algorithm also showed a good prediction ability for MOF systems [56,57,58].

**Figure 4:**
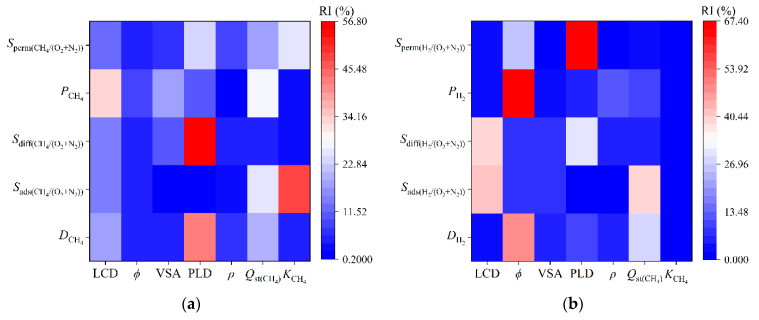
Relative importance values of the seven descriptors predicted by the RF algorithm for: (**a**) CH_4_/O_2_ + N_2_; and (**b**) H_2_/O_2_ + N_2_.

Based on the discussion above, RF was further used to estimate the influences of the seven descriptors of the MOFs on the performances of the MOF adsorbents and membranes. The results are shown in Figure 4. The relative importance (RI) analysis showed that in the CH_4_/N_2_ + O_2_ system (Figure 4a), the Henry’s constant of *S*_ads(CH4/O2+N2)_ had the highest importance (about 49%, more than two times that of *Q*_st_), and the RI order was *K*_CH4_ > *Q*_st(CH4)_ > LCD > *ϕ* > *ρ* > PLD ≥ VSA. This is consistent with the conclusion of Cai et al. [59] that the Henry’s constant is the key descriptor of the trade-offs of C_1_, C_2_, and C_3_. In addition, the RF model quantitatively showed that PLD was the first important descriptor of the CH_4_ diffusion coefficient and diffusion selectivity, which was a similar conclusion to that of Yang et al. [60]. For *P*_CH4_, the RI values of LCD and *Q*_st(CH4)_ of the MOFMs were large, with values of 33.50% and 28.14%, respectively. At the same time, the RF model analysis showed that *K*_CH4_ was the most important descriptor of *S*_perm_, and LCD was the second most important. In the H_2_/N_2_ + O_2_ system (Figure 4b), LCD was the most important influencing factor for both *S*_ads_ and *S*_diff_, with an RI of about 40%; *ϕ* was the most important descriptor of *P*, with a large RI (about 5.6 times that of *ρ*), and the RI order was *ϕ* > *ρ* > *Q*_st(H2)_ > PLD > VSA ≥ LCD > *K*_H2_. In addition, *ϕ* was an important factor for *S*_perm_, and the change of PLD (RI of about 67%) could also affect *S*_perm_. These results showed that the RF algorithm could accurately predict the performance indicators of the two systems by using the relevant descriptors of the MOFs and quantitatively determine the importance of the descriptors. This is conducive to screening candidate materials from a large number of MOFs to further guide experiments.

### 3.4. Top-Performing MOFs and MOFMs

**Figure 5:**
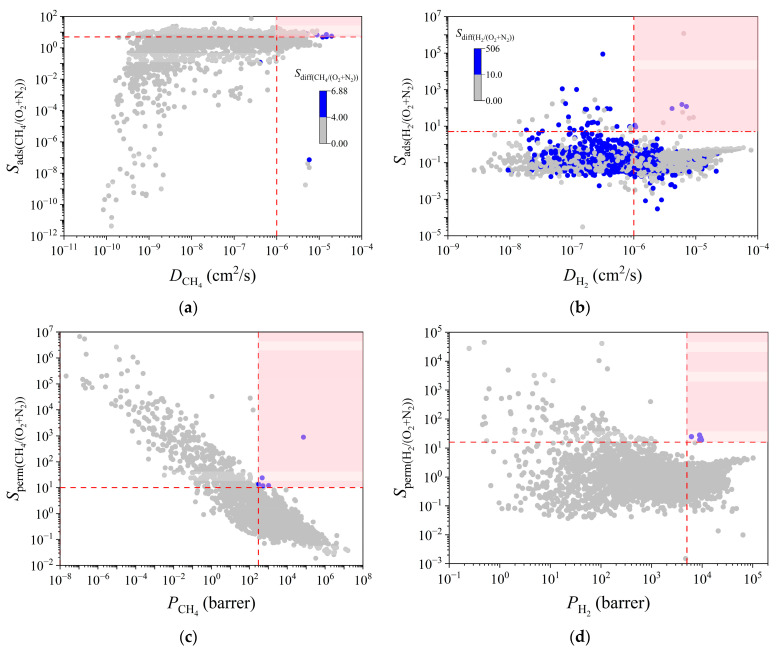
(**a**,**b**) Selectivity *S*_ads_ vs. diffusion coefficient *D* and (**c**,**d**) permeation selectivity *S*_perm_ vs. permeability *P* for CH_4_/O_2_ + N_2_ and H_2_/O_2_ + N_2_ in 6013 CoRE-MOFs. The color coding represents the diffusion selectivity *S*_diff_.

In order to select MOFs and MOFMs with excellent performances for different systems, we used the limiting conditions shown in Table S4 to determine the best MOFs of the two systems as well as *D*, *S*_ads_, *S*_diff_, *P*, and *S*_perm_ of each adsorbate gas molecule in the MOFMs. Five optimal MOFs and five optimal MOFMs were screened for each system, which are listed in Table 2. In Figure 5a,b, the blue dots in the pink shaded part represent the highest-potential MOFs, which showed the best combination of adsorption and diffusion selectivity and had high *D*_CH4_ or *D*_H2_ values simultaneously. However, it was further found that most of the MOFs in Table S4 had large *S*_diff(H2/(O2+N2))_ values, but only when the diffusion coefficient of H_2_ was larger, was the MOF more conducive to separation. Among the five materials selected for the CH_4_/N_2_ + O_2_ mixed system, the *S*_ads(CH4/O2+N2)_, *S*_diff(CH4/O2+N2)_, and *D*_CH4_ values of the ITAHEQ MOF were 7.61, 6.79, and 4.84 × 10^−6^ cm^2^/s, respectively, which were the largest adsorption and diffusion selectivity values of the materials considered. In addition, *S*_diff(CH4/O2+N2)_ was generally lower than *S*_ads(CH4/O2+N2)_, which also meant that the diffusion property of CH_4_ was the key performance metric for the MOFs to capture low-concentration CH_4_ from N_2_ and O_2_ during dynamic adsorption and membrane separation. Moreover, some MOFs in Figure 5a,c with very small pores only could adsorb single special gas molecules, leading to high selectivity. However, these MOFs also possess very low loading or permeability, so they are still not good candidates. This was consistent with the previous RI analysis. PLD is the key descriptor to control *D*_CH4_ and *S*_diff(CH4/O2+N2)_. Likewise, Sumer [61] pointed out that CH_4_ has a stronger adsorption capacity than N_2_, and in his work, the better performing MOFs screened showed high adsorption selectivity for CH_4_ (1.3–9) over weak diffusivity selectivity for N_2_ (1–4), which makes them methane-selective membranes. Qiao [26] also showed that diffusion is not only influenced by the size of gas molecules but also by gas–framework interactions, and *S*_diff_(N_2_/CH_4_) is much larger than *S*_ads_(N_2_/CH_4_). The separation is controlled by diffusion, with methane diffusion being much slower and N_2_/CH_4_ separation being driven by diffusion. In the H_2_/N_2_ + O_2_ system, the analysis of the optimal MOFs showed that the LCD range was very concentrated, ranging from 2.89 to 2.98 Å. This was because the N_2_ and O_2_ molecules in the LCD range could not enter the channel due to the large molecular dynamic diameters, which significantly improved the H_2_ separation performance. At the same time, according to the analysis of the RI of the descriptors calculated by the RF algorithm, LCD is the key descriptor to control H_2_/N_2_ + O_2_ separation. This showed that LCD with a kinetic diameter close to the H_2_ diameter was the key for capturing H_2_ molecules from the air and achieving a good separation effect, which provides effective theoretical guidance for the design and synthesis of new MOFs. The optimal MOFMs of the two systems are shown with blue dots in Figure 5c,d, with the highest *P* and *S*_perm_ values. These optimal MOFMs often have narrow channel structures, especially in H_2_/N_2_ + O_2_. LCD was mainly concentrated in the range of 2.74–2.83 Å, and PLD was in the range of 2.44–2.52 Å. For the optimal MOFMs for CH_4_ separation, the PLD also ranged from 2.85–3.49 Å, but the values were higher than those for the H_2_ separation membranes because the size of the CH_4_ molecule is larger than that of H_2_. Thus, CH_4_ requires larger pores to pass through the membrane. Since VSA was calculated by GCMC using N_2_ gas molecules as probes, when the pore diameter was smaller than the N_2_ molecular diameter, VSA was displayed as 0. Therefore, the VSA of almost all the optimal MOFMs was 0. These MOFMs showed excellent separation performances, and their *P*_H2_ values were between 6000 and 10,000 barrer. *S*_perm_ was also above 10, which is suitable for H_2_ membrane separation applications.

### 3.5. Design Strategies of MOFs and MOFMs with High Performances

**Figure 6:**
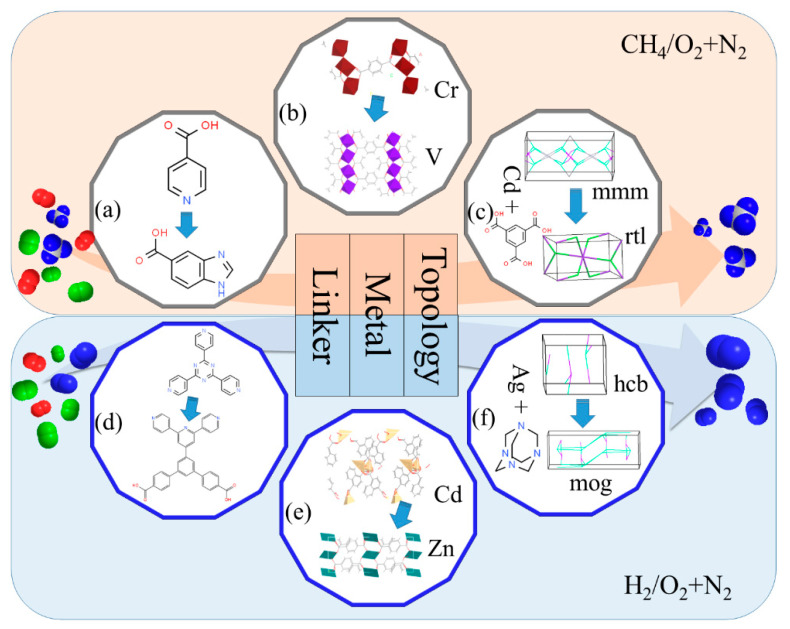
Three design strategies for boosting the separation of (**a**–**c**) CH_4_/O_2_ + N_2_; and (**d**–**f**) H_2_/O_2_ + N_2_.

In order to further guide the experiment and design and synthesize new MOF adsorbents or membranes with excellent performance, this section discusses a series of design strategies for a series of new hypothetical MOF materials based on ml and HTCS predictions. Firstly, 10 pairs of materials (20 pairs in total) are selected for each system. Each pair contains one MOF with excellent performance and one MOF with poor performance, but only one MOF composition has changed between them, such as topology, connector, or metal center, as shown in Table S5. Based on this, we propose three design strategies to facilitate the capture of CH_4_ or H_2_ from the air, as shown in Figure 6. Three pairs of representative design strategies are selected for each system, similar to the previous research work [34,62]. Among them, Figure 6a–c show three strategies for the CH_4_/O_2_ + N_2_ system, and Figure 6d–f show three strategies for H_2_/O_2_ + N_2_ system. Figure 6a,d display that changes in organic linkers result in different separation effects. Combined with RI, *K*_CH4,_ and LCD, their adsorption selectivity and permeability were greatly affected. In Figure 6a for MOFMs, different organic connectors change the size of the channel, making the channel more compact. The LCD is reduced from 6.906 Å to 5.769 Å. Both LCD and PLD are closer to the dynamic diameter (3.8 Å) of CH_4_, thus enhancing the separation effect of CH_4_. For MOF adsorbent, the Henry’s constant of MOF with excellent performance is relatively large (1.36 × 10^−5^ > 8.07 × 10^−6^), plus with above influence of LCD, CH_4_ can be adsorbed preferentially. Figure 6b,e have the same topology and organic links but different metal centers. In Figure 6b, MOF with good performance takes V as the metal center. Since the atomic radius is similar to that of Cr, but the metal activity of V is stronger than that of Cr, it has better adsorption, separation, or permeability. Figure 6e depicts that the atomic radius of metal Zn is smaller than that of Cd. Therefore, the regulation of the metal center affects the pore size of MOF and then affects the properties. *S*_ads_, *S*_diff_, and *S*_perm_ all increased by 60%~300%. Therefore, finding suitable metal nodes for a certain system can be considered an effective strategy to improve the separation performance. Figure 6c,f show the same metal centers and organic linkers, but different topologies lead to different separation effects. Among them, Figure 6c shows two different topological structures (mmm and rtl) assembled by the Cd-based metal center and 1,3,5-benzenetricarboxylic acid. Because the pore channels formed by different topological networks have different pore diameters and shapes, the diffusion coefficient is increased by 79%, and the gas permselectivity is increased by 120%. Figure 6f shows two different topologies (hcb and mog) assembled by the Ag-based metal center and 1,3,5,7-tetrazatricyclo[3.3.1.13,7]decane. The pore shape is more complex and has better performance of H_2_ separation. Therefore, for different systems, better MOF adsorbents or membranes can be obtained or designed by adjusting more appropriate topological structures, connectors, or metal centers.

## 4. Conclusions

In this work, the adsorption separation performances of 6013 CoRE-MOF adsorbents and membranes for CH_4_/N_2_ + O_2_ and H_2_/N_2_ + O_2_ mixtures were simulated using large-scale computational screening and ML. First, the close relationship between *D*(*P*) and PLD in both systems was initially examined by univariate analysis, and the quantitative relationships log *D* = aPLD − b and log *P* = cPLD − d were established. Then, three ML methods, TOPT, DT, and RF, were used to predict the performance indices of each system. The analysis of the results showed that the model prediction accuracies were ranked as RF ≥ TPOT > DT. Moreover, the feature importance was determined by the RF algorithm, and the RI ranking for the adsorption selectivity prediction of CH_4_ was *K*_CH4_ > *Q*_st(CH4)_ > LCD > *ϕ* > *ρ* > PLD ≥ VSA. Nevertheless, the descriptor that had the greatest impact on *D*_CH4_ and *S*_diff(CH4/O2+N2)_ was PLD. LCD and *Q*_st(CH4)_ were the two important descriptors of *P* and *S*_perm_. In the H_2_/N_2_ + O_2_ system, LCD had the largest RI for the performance of the H_2_ adsorbent, while for P, the RI ranking was *ϕ* > *ρ* > *Q*_st(H2)_ > PLD > VSA ≥ LCD > *K*_H2_. Furthermore, five optimal MOFs and five optimal MOFMs were screened for each of the two systems. The analysis of the optimal MOFs also further verified that the diffusivity of CH_4_ is a key indicator to test in dynamic adsorption or membrane separation processes, and an LCD close to the kinetic diameter of H_2_ is an overriding condition to achieve the separation of H_2_ from air. Finally, three types of design strategies, tuned topology, alternative metal nodes, and organic linkers, were proposed to effectively facilitate the capture of low-concentration CH_4_ or H_2_ from the air for specific applications. This study may provide an effective guide for experimental researchers to find MOFs/MOFMs for capturing energy gases (CH_4_ and H_2_) from air and can lead to new research ideas for applications involving coal mine spent air and hydrogen leakage.

## Figures and Tables

**Table 1:** Evaluation of three ML algorithms for the systems of CH_4_/(O_2_ + N_2_) and H_2_/(O_2_ + N_2_).

System	Performance Indicators	Machine Learning Methods	Training Set			Test Set		
			*R*	MAE	RMSE	*R*	MAE	RMSE
CH_4_/(O_2_ + N_2_)	*D*_CH4_	TPOT	0.93	1.20	3.44	0.88	1.48	3.15
		DT	0.86	1.98	4.70	0.80	1.87	3.96
		RF	0.91	1.46	3.87	0.85	2.08	4.42
	*S*_ads(CH4/O2+N2)_	TPOT	0.98	0.21	0.56	0.95	0.39	0.72
		DT	0.96	0.39	0.72	0.93	0.52	0.86
		RF	0.99	0.15	0.42	0.96	0.36	0.64
	*S*_diff(CH4/O2+N2)_	TPOT	0.90	0.10	0.19	0.84	0.14	0.22
		DT	0.85	0.15	0.23	0.78	0.16	0.25
		RF	0.91	0.10	0.18	0.85	0.14	0.21
	log(*P*_CH4_)	TPOT	0.98	0.35	0.47	0.97	0.37	0.52
		DT	0.99	0.14	0.37	0.98	0.29	0.47
		RF	0.99	0.21	0.32	0.99	0.26	0.36
	log(*S*_perm(CH4/O2+N2)_)	TPOT	0.97	0.19	0.34	0.96	0.21	0.38
		DT	0.97	0.19	0.37	0.95	0.21	0.36
		RF	0.98	0.14	0.29	0.97	0.17	0.31
H_2_/(O_2_ + N_2_)	*D*_H2_	TPOT	0.98	4.58	9.36	0.95	6.36	10.81
		DT	0.96	8.01	12.94	0.92	8.25	13.81
		RF	0.99	3.03	5.50	0.95	6.30	10.73
	log(*S*_ads(H2/O2+N2)_)	TPOT	0.97	0.03	0.10	0.95	0.06	0.14
		DT	0.97	0.06	0.11	0.92	0.08	0.17
		RF	0.98	0.03	0.08	0.95	0.06	0.13
	log(*S*_diff(H2/O2+N2)_)	TPOT	0.76	0.12	0.18	0.71	0.13	0.19
		DT	0.71	0.13	0.20	0.65	0.13	0.21
		RF	0.78	0.12	0.17	0.73	0.12	0.19
	*P*_H2_	TPOT	0.98	556.09	1226.85	0.94	1179.56	2367.19
		DT	0.97	961.07	1862.85	0.91	1497.22	2896.62
		RF	0.98	694.80	1418.32	0.93	1245.29	2498.44
	*S*_perm(H2/O2+N2)_	TPOT	0.97	0.13	0.19	0.96	0.17	0.25
		DT	0.97	0.13	0.20	0.94	0.19	0.29
		RF	0.99	0.07	0.11	0.96	0.15	0.23

**Table 2:** Best MOFs and MOFMs.

Application	Systems	CSD Code	LCD (Å)	*ϕ*	VSA (m^2^/cm^3^)	PLD (Å)	*ρ* (kg/m^3^)	*Q*_st_ (kJ/mol)	*K* (mol/kg/Pa)	*D*_CH4_ (10^−6^ cm^2^/s)	S_ads_	*S*_diff_
MOFs	CH_4_/N_2_ + O_2_	ITAHEQ	4.67	0.15	105.10	4.13	1774.31	25.29	1.26 × 10^−5^	4.84	7.61	6.79
		QATLEE	4.25	0.31	289.95	4.04	2185.04	25.25	2.28 × 10^−5^	19.58	5.57	6.88
		XEJVOZ	5.05	0.16	274.91	4.70	2255.03	24.01	1.35 × 10^−5^	14.76	7.05	4.93
		FUDQIF	4.38	0.37	261.53	3.86	1573.76	27.47	7.58 × 10^−5^	8.92	6.64	4.69
		REGJIW	4.22	0.30	239.00	4.03	2185.50	24.82	1.73 × 10^−5^	14.90	5.11	5.98
	H_2_/N_2_ + O_2_	ja4050828	2.89	0.04	0.00	2.41	1779.43	4.57	3.3 × 10^−9^	5.97	153.95	504.05
		ja4044642_si_002	2.90	0.04	0.00	2.43	1773.83	4.60	3.6 × 10^−9^	7.08	119.50	87.59
		ja403810k_si_003	2.92	0.04	0.00	2.44	1764.65	4.80	4.2 × 10^−9^	4.14	91.54	161.42
		UMEMAB	2.97	0.09	0.00	2.51	2543.92	6.19	8.8 × 10^−9^	1.04	11.22	19.43
		IFUDAO	2.98	0.12	0.00	2.84	1772.35	6.11	1.9 × 10^−9^	1.08	9.03	17.63
**Application**	**Systems**	**CSD Code**	**LCD** **(Å)**	***ϕ***	**VSA** **(m^2^/cm^3^)**	**PLD** **(Å)**	***ρ* (kg/m^3^)**	***Q*_st_** **(kJ/mol)**	***K*** **(mol/kg/Pa)**	***P* (barrer) ^a^**	***S*_perm_**	
MOFMs	CH_4_/N_2_ + O_2_	GOJRED	7.79	0.34	362.41	2.85	1251.40	40.28	4.47 × 10^−4^	72177.49	892.96	
		ZIHTEP	4.35	0.17	108.53	3.49	1109.96	20.74	3.85 × 10^−6^	475.23	23.97	
		XORGUI	4.07	0.35	58.28	3.22	1719.10	26.83	1.49 × 10^−5^	312.12	13.53	
		LULJAE	3.65	0.30	57.51	3.28	1414.94	23.86	8.18 × 10^−6^	1038.17	11.98	
		YAYPAR	3.81	0.18	4.86	3.12	2963.52	25.23	4.16 × 10^−6^	521.57	11.72	
	H_2_/N_2_ + O_2_	WENSIS	2.75	0.25	0.00	2.44	1625.34	16.23	9.07 × 10^−7^	8900.57	28.15	
		IDAZEU	2.74	0.25	0.00	2.46	2245.15	16.40	6.73 × 10^−7^	9354.21	22.65	
		IQUNAJ01	2.83	0.25	0.00	2.52	1719.74	16.28	8.85 × 10^−7^	9726.40	19.04	
		IQUNAJ	2.83	0.24	0.00	2.52	1726.63	16.44	8.63 × 10^−7^	8950.93	19.30	
		YAFGAP	2.75	0.24	0.00	2.44	2321.42	14.40	3.86 × 10^−7^	6142.86	24.84	

^a^ 1 barrer = 3.348 × 10^–16^ mol·m/(m^2^·s·Pa) = 10^−10^ cm^3^ (STP) cm/(cm^2^·s·cmHg).

## Supplementary Materials

The supplementary material can be downloaded at: https://www.mdpi.com/article/10.3390/membranes12090830/s1. Figure S1: The relationship between the descriptors of MOFs with adsorbents performance in CH_4_/N_2_ + O_2_; Figure S2: The relationship between the descriptors of MOFs with adsorbents performance in H_2_/N_2_ + O_2_; Figure S3: The relationship between the descriptors of MOFMs with adsorbents performance in CH_4_/N_2_ + O_2_; Figure S4: The relationship between the descriptors of MOFMs with adsorbents performance in H_2_/N_2_ + O_2_; Figure S5: Diffusion coefficient D and Permeability P versus PLD for N_2_ and O_2_ in 6013 CORE-MOFs; Figure S6: Tree-based pipeline optimization tool; Figure S7: Decision tree; Figure S8: Random forest; Figure S9: k-fold cross validation; Table S1: Lennard–Jones parameters of MOFs; Table S2: Lennard–Jones parameters and charges of adsorbates; Table S3: Kinetic diameter of CH_4_, N_2_, O_2_ and H_2_; Table S4: The constraints for screening the best MOFs/MOFMs under each system; Table S5: Design Strategies of MOFs and MOFMs with high performance. References [48,63,64,65,66] are cited in the supplementary materials.

## References

[1] Li W., Xia X., Li S. (2019). Large-scale evaluation of cascaded adsorption heat pumps based on metal/covalent–organic frameworks. J. Mater. Chem. A.

[2] Lin R.-B., Xiang S., Zhou W., Chen B. (2020). Microporous metal-organic framework materials for gas separation. Chem.

[3] Van Vleet M.J., Weng T.T., Li X.Y., Schmidt J.R. (2018). In situ, time-resolved, and mechanistic studies of metal-organic framework nucleation and growth. Chem. Rev..

[4] Lu Y., Zhang H., Chan J.Y., Ou R., Zhu H., Forsyth M., Marijanovic E.M., Doherty C.M., Marriott P.J., Holl M.M.B. (2019). Homochiral MOF–polymer mixed matrix membranes for efficient separation of chiral molecules. Angew. Chem. Int. Ed..

[5] Wang M., Wang T., Cai P., Chen X. (2019). Nanomaterials discovery and design through machine learning. Small Methods.

[6] Shi Z., Liang H., Yang W., Liu J., Liu Z., Qiao Z. (2020). Machine learning and in silico discovery of metal-organic frameworks: Methanol as a working fluid in adsorption-driven heat pumps and chillers. Chem. Eng. Sci..

[7] Yuan X., Li L., Shi Z., Liang H., Li S., Qiao Z. (2022). Molecular-fingerprint machine-learning-assisted design and prediction for high-performance MOFs for capture of NMHCs from air. Adv. Powder Mater..

[8] He T., Pachfule P., Wu H., Xu Q., Chen P. (2016). Hydrogen carriers. Nat. Rev. Mater..

[9] Feng L., Wang Y., Zhang K., Wang K.-Y., Fan W., Wang X., Powell J.A., Guo B., Dai F., Zhang L. (2019). Molecular pivot-hinge installation to evolve topology in rare-earth metal-organic frameworks. Angew. Chem. Int. Ed..

[10] Luo L., Lo W.S., Si X., Li H., Wu Y., An Y., Zhu Q., Chou L.Y., Li T., Tsung C.K. (2019). Directional engraving within single crystalline metal-organic framework particles via oxidative linker cleaving. J. Am. Chem. Soc..

[11] Zhang F., Liu Y., Lei J., Wang S., Ji X., Liu H., Yang Q. (2019). Metal-organic-framework-derived carbon nanostructures for site-specific dual-modality photothermal/photodynamic thrombus therapy. Adv. Sci..

[12] Liu D., Lu K., Poon C., Lin W. (2014). Metal-organic frameworks as sensory materials and imaging agents. Inorg. Chem..

[13] Chang M., Ren J., Yang Q., Liu D. (2021). A robust calcium-based microporous metal-organic framework for efficient CH_4_/N_2_ separation. Chem. Eng. J..

[14] Xu G.J., Meng Z.S., Liu Y.Z., Guo X.J., Deng K.M., Ding L.F., Lu R.F. (2019). Porous MOF-205 with multiple modifications for efficiently storing hydrogen and methane as well as separating carbon dioxide from hydrogen and methane. Int. J. Energ. Res..

[15] Kang Z., Xue M., Fan L., Huang L., Guo L., Wei G., Chen B., Qiu S. (2014). Highly selective sieving of small gas molecules by using an ultra-microporous metal–organic framework membrane. Energ. Environ. Sci..

[16] Hou Q., Wu Y., Zhou S., Wei Y., Caro J., Wang H. (2019). Ultra-tuning of the aperture size in stiffened ZIF-8_Cm frameworks with mixed-linker strategy for enhanced CO_2_/CH_4_ separation. Angew. Chem. Int. Ed..

[17] Gulbalkan H.C., Haslak Z.P., Altintas C., Uzun A., Keskin S. (2022). Assessing CH_4_/N_2_ separation potential of MOFs, COFs, IL/MOF, MOF/Polymer, and COF/Polymer composites. Chem. Eng. J..

[18] Belmabkhout Y., Mouttaki H., Eubank J.F., Guillerm V., Eddaoudi M. (2014). Effect of pendant isophthalic acid moieties on the adsorption properties of light hydrocarbons in HKUST-1-like tbo-MOFs: Application to methane purification and storage. RSC Adv..

[19] Kang Z., Fan L., Sun D. (2017). Recent advances and challenges of metal–organic framework membranes for gas separation. J. Mater. Chem. A.

[20] Fan H., Peng M., Strauss I., Mundstock A., Meng H., Caro J. (2021). MOF-in-COF molecular sieving membrane for selective hydrogen separation. Nat. Commun..

[21] Yang L., Qian S., Wang X., Cui X., Chen B., Xing H. (2020). Energy-efficient separation alternatives: Metal-organic frameworks and membranes for hydrocarbon separation. Chem. Soc. Rev..

[22] Zhang Y., Feng X., Yuan S., Zhou J., Wang B. (2016). Challenges and recent advances in MOF-polymer composite membranes for gas separation. Inorg. Chem. Front..

[23] Liu G., Chernikova V., Liu Y., Zhang K., Belmabkhout Y., Shekhah O., Zhang C., Yi S., Eddaoudi M., Koros W.J. (2018). Mixed matrix formulations with MOF molecular sieving for key energy-intensive separations. Nat. Mater..

[24] Wang Y., Fin H., Ma Q., Mo K., Mao H., Feldhoff A., Cao X., Li Y., Pan F., Jiang Z. (2020). A MOF glass membrane for gas separation. Angew. Chem. Int. Ed..

[25] Watanabe T., Sholl D.S. (2012). Accelerating applications of metal-organic frameworks for gas adsorption and separation by computational screening of materials. Langmuir.

[26] Qiao Z., Peng C., Zhou J., Jiang J. (2016). High-throughput computational screening of 137953 metal–organic frameworks for membrane separation of a CO_2_/N_2_/CH_4_ mixture. J. Mater. Chem. A.

[27] Qiao Z., Xu Q., Jiang J. (2018). Computational screening of hydrophobic metal–organic frameworks for the separation of H_2_S and CO_2_ from natural gas. J. Mater. Chem. A.

[28] McIntyre S.M., Shan B., Wang R., Zhong C., Liu J., Mu B. (2018). Monte carlo simulations to examine the role of pore structure on ambient air separation in metal–organic frameworks. Ind. Eng. Chem. Res..

[29] Qiao Z., Li L., Li S., Liang H., Zhou J., Snurr R.Q. (2021). Molecular fingerprint and machine learning to accelerate design of high-performance homochiral metal-organic frameworks. AIChE J..

[30] Shi Z., Yuan X., Yan Y., Tang Y., Li J., Liang H., Tong L., Qiao Z. (2021). Techno-economic analysis of metal–organic frameworks for adsorption heat pumps/chillers: From directional computational screening, machine learning to experiment. J. Mater. Chem. A.

[31] Anderson R., Rodgers J., Argueta E., Biong A., Gómez-Gualdrón D.A. (2018). Role of pore chemistry and topology in the CO_2_ capture capabilities of MOFs: From molecular simulation to machine learning. Chem. Mater..

[32] Moosavi S.M., Nandy A., Jablonka K.M., Ongari D., Janet J.P., Boyd P.G., Lee Y., Smit B., Kulik H.J. (2020). Understanding the diversity of the metal-organic framework ecosystem. Nat. Commun..

[33] Li H., Li L., Lin R.-B., Zhou W., Zhang Z., Xiang S., Chen B. (2019). Porous metal-organic frameworks for gas storage and separation: Status and challenges. EnergyChem.

[34] Yan Y., Shi Z., Li H., Li L., Yang X., Li S., Liang H., Qiao Z. (2022). Machine learning and in-silico screening of metal–organic frameworks for O_2_/N_2_ dynamic adsorption and separation. Chem. Eng. J..

[35] Tang H., Xu Q., Wang M., Jiang J. (2021). Rapid screening of metal-organic frameworks for propane/propylene separation by synergizing molecular simulation and machine learning. ACS Appl. Mater. Inter..

[36] Rosen A.S., Iyer S.M., Ray D., Yao Z., Aspuru-Guzik A., Gagliardi L., Notestein J.M., Snurr R.Q. (2021). Machine learning the quantum-chemical properties of metal–organic frameworks for accelerated materials discovery. Matter.

[37] Moosavi S.M., Chidambaram A., Talirz L., Haranczyk M., Stylianou K.C., Smit B. (2019). Capturing chemical intuition in synthesis of metal-organic frameworks. Nat. Commun..

[38] Azar A.N.V., Velioglu S., Keskin S. (2019). Large-scale computational screening of metal organic framework (MOF) membranes and MOF-based polymer membranes for H_2_/N_2_ separations. ACS Sustain. Chem. Eng..

[39] Qiao Z., Xu Q., Jiang J. (2018). High-throughput computational screening of metal-organic framework membranes for upgrading of natural gas. J. Membr. Sci..

[40] Bai X., Shi Z., Xia H., Li S., Liu Z., Liang H., Liu Z., Wang B., Qiao Z. (2022). Machine-Learning-Assisted High-Throughput computational screening of Metal-Organic framework membranes for hydrogen separation. Chem. Eng. J..

[41] Skoulidas A.I., Sholl D.S. (2005). Self-diffusion and transport diffusion of light gases in metal-organic framework materials assessed using molecular dynamics simulations. J. Phys. Chem. B.

[42] Erucar I., Keskin S. (2016). Computational assessment of MOF membranes for CH_4_/H_2_ separations. J. Membr. Sci..

[43] Adatoz E., Avci A.K., Keskin S. (2015). Opportunities and challenges of MOF-based membranes in gas separations. Sep. Purif. Technol..

[44] Avci G., Velioglu S., Keskin S. (2018). High-throughput screening of MOF adsorbents and membranes for H_2_ purification and CO_2_ capture. ACS Appl. Mater. Inter..

[45] Chung Y.G., Camp J., Haranczyk M., Sikora B.J., Bury W., Krungleviciute V., Yildirim T., Farha O.K., Sholl D.S., Snurr R.Q. (2014). Computation-ready, experimental metal–organic frameworks: A tool to enable high-throughput screening of nanoporous crystals. Chem. Mater..

[46] Willems T.F., Rycroft C.H., Kazi M., Meza J.C., Haranczyk M. (2012). Algorithms and tools for high-throughput geometry-based analysis of crystalline porous materials. Micropor. Mesopor. Mat..

[47] Zhou Y.P., Wei L.F., Yang J., Sun Y., Zhou L. (2005). Adsorption of oxygen on superactivated carbon. J. Chem. Eng. Data.

[48] Potoff J.J., Siepmann J.I. (2001). Vapor-liquid equilibria of mixtures containing alkanes, carbon dioxide, and nitrogen. AIChE J..

[49] Garberoglio G., Skoulidas A.I., Johnson J.K. (2005). Adsorption of gases in metal organic materials: Comparison of simulations and experiments. J. Phys. Chem. B.

[50] Qiao Z., Xu Q., Cheetham A.K., Jiang J. (2017). High-throughput computational screening of metal–organic frameworks for thiol capture. J. Phys. Chem. C.

[51] Ewald P.P. (1921). Die Berechnung optischer und elektrostatischer Gitterpotentiale. Ann. Phys..

[52] Hantal G., Jedlovszky P., Hoang P.N.M., Picaud S. (2007). Calculation of the adsorption isotherm of formaldehyde on ice by grand canonical Monte Carlo simulation. J. Phys. Chem. C.

[53] Pedregosa F., Varoquaux G., Gramfort A., Michel V., Thirion B., Grisel O., Blondel M., Prettenhofer P., Weiss R., Dubourg V. (2011). Scikit-learn: Machine learning in python. J. Mach. Learn. Res..

[54] Wilmer C.E., Farha O.K., Bae Y.-S., Hupp J.T., Snurr R.Q. (2012). Structure-property relationships of porous materials for carbon dioxide separation and capture. Energ. Environ. Sci..

[55] Keskin S., Sholl D.S. (2009). Efficient methods for screening of metal organic framework membranes for gas separations using atomically detailed models. Langmuir.

[56] Yuan X., Deng X., Cai C., Shi Z., Liang H., Li S., Qiao Z. (2021). Machine learning and high-throughput computational screening of hydrophobic metal-organic frameworks for capture of formaldehyde from air. Green Energy Environ..

[57] Deng X., Yang W., Li S., Liang H., Shi Z., Qiao Z. (2020). Large-scale screening and machine learning to predict the computation-ready, experimental metal-organic frameworks for CO_2_ capture from air. Appl. Sci..

[58] Wu X., Xiang S., Su J., Cai W. (2019). Understanding quantitative relationship between methane storage capacities and characteristic properties of metal-organic frameworks based on machine learning. J. Phys. Chem. C.

[59] Cai C., Li L., Deng X., Li S., Liang H., Qiao Z. (2020). Machine learning and high-throughput computational screening of metal-organic framework for separation of methane/ethane/propane. Acta Chim. Sin..

[60] Yang W., Liang H., Peng F., Liu Z., Liu J., Qiao Z. (2019). Computational screening of metal-organic framework membranes for the separation of 15 gas mixtures. Nanomaterials.

[61] Sumer Z., Keskin S. (2017). Adsorption- and membrane-based CH_4_/N_2_ separation performances of MOFs. Ind. Eng. Chem. Res..

[62] Tang H., Jiang J. (2021). In silico screening and design strategies of ethane-selective metal-organic frameworks for ethane/ethylene separation. AIChE J..

[63] Rappé A.K., Casewit C.J., Colwell K.S., Goddard W.A., Skiff W.M. (1992). Uff a full periodic table force field for molecular mechanics and molecular dynamics simulations. J. Am. Chem. Soc..

[64] Stoll J., Vrabec J., Hasse H. (2003). Vapor-liquid equilibria of mixtures containing nitrogen, oxygen, carbon dioxide, and ethane. AIChE J..

[65] Martin M.G., Siepmann J.I. (1998). Transferable Potentials for Phase Equilibria. 1. United-Atom Description of n-Alkanes. J. Phys. Chem. B.

[66] Shah M.S., Tsapatsis M., Siepmann J.I. (2015). Development of the Transferable Potentials for Phase Equilibria Model for Hydrogen Sulfide. J. Phys. Chem. B.

