# Supplementary material for: Combining Computational Screening and Machine Learning to Predict Metal–Organic Framework Adsorbents and Membranes for Removing CH4 or H2 from Air
Source: Membranes (Basel). 2022 Aug 25;12(9):830. doi: 10.3390/membranes12090830 (PMC9503901; doi:10.3390/membranes12090830)
Supplement: Supplementary file 1 [file membranes-12-00830-s001.zip › membranes-1854793-supplementary.pdf]

Supplementary Materials

Combining computational screening and machine learning to predict metal–organic framework adsorbents and membranes for removing CH_4 or H_2 from air

Huilin Li [1], Cuimiao Wang [1], Yue Zeng [1], Dong Li [1], Yaling Yan [1,*], Xin Zhu [1,*] and Zhiwei Qiao [1,2,*]

1 Guangzhou Key Laboratory for New Energy and Green Catalysis, School of Chemistry and Chemical Engineering, Guangzhou University, Guangzhou 510006, China

2 Joint Institute of Guangzhou University & Institute of Corrosion Science and Technology, Guangzhou University, Guangzhou 510006, China

Correspondence: zqiao@gzhu.edu.cn; yaling@gzhu.edu.cn; zhux@gzhu.edu.cn.

Table Contents

Table S1 Lennard–Jones parameters of MOFs S2

Table S2 Lennard–Jones parameters and charges of adsorbates S3

Table S3 Kinetic diameter of CH_4, N_2, O_2 and H_2 S3

Table S4 The constraints for screening the best MOFs/MOFMs under each system S12

Table S5 Design Strategies of MOFs and MOFMs with high performance S12

Figure Contents

Figure S1 The relationship between the descriptors of MOFs with adsorbents performance in CH_4/N_2+O_2 S4

Figure S2 The relationship between the descriptors of MOFs with adsorbents performance in H_2/N_2+O_2 S5

Figure S3 The relationship between the descriptors of MOFMs with adsorbents performance in CH_4/N_2+O_2 S6

Figure S4 The relationship between the descriptors of MOFMs with adsorbents performance in H_2/N_2+O_2 S7

Figure S5 Diffusion coefficient *D* and Permeability *P versus* PLD for N_2 and O_2 in 6013 CORE-MOFs S8

Figure S6 Tree-based pipeline optimization tool S9

Figure S7 Decision tree S9

Figure S8 Random forest S10

Figure S9 k-fold cross validation S11

Others

Machine learning methods S9

k-fold cross validation S11

Evaluation indicators of ML algorithms S11

References S27

Table S1. Lennard-Jones parameters of MOFs[1].

Atom	ε/k_B [K]	σ [Å]	Atom	ε/k_B [K]	σ [Å]	Atom	ε/k_B [K]	σ [Å]
Ac	16.60	3.10	Ge	190.69	3.81	Po	163.52	4.20
Ag	18.11	2.80	Gd	4.53	3.00	Pr	5.03	3.21
Al	254.09	4.01	H	22.14	2.57	Pt	40.25	2.45
Am	7.04	3.01	Hf	36.23	2.80	Pu	8.05	3.05
Ar	93.08	3.45	Hg	193.71	2.41	Ra	203.27	3.28
As	155.47	3.77	Ho	3.52	3.04	Rb	20.13	3.67
At	142.89	4.23	I	170.57	4.01	Re	33.21	2.63
Au	19.62	2.93	In	301.39	3.98	Rh	26.67	2.61
B	90.57	3.64	Ir	36.73	2.53	Rn	124.78	4.25
Ba	183.15	3.30	K	17.61	3.40	Ru	28.18	2.64
Be	42.77	2.45	Kr	110.69	3.69	S	137.86	3.59
Bi	260.63	3.89	La	8.55	3.14	Sb	225.91	3.94
Bk	6.54	2.97	Li	12.58	2.18	Sc	9.56	2.94
Br	126.29	3.73	Lu	20.63	3.24	Se	146.42	3.75
C	52.83	3.43	Lr	5.53	2.88	Si	202.27	3.83
Ca	119.75	3.03	Md	5.53	2.92	Sm	4.03	3.14
Cd	114.72	2.54	Mg	55.85	2.69	Sn	285.28	3.91
Ce	6.54	3.17	Mn	6.54	2.64	Sr	118.24	3.24
Cf	6.54	2.95	Mo	28.18	2.72	Ta	40.75	2.82
Cl	114.21	3.52	N	34.72	3.26	Tb	3.52	3.07
Cm	6.54	2.96	Na	15.09	2.66	Tc	24.15	2.67
Co	7.04	2.56	Ne	21.13	2.66	Te	200.25	3.98
Cr	7.55	2.69	Nb	29.69	2.82	Th	13.08	3.03
Cu	2.52	3.11	Nd	5.03	3.18	Ti	8.55	2.83
Cs	22.64	4.02	No	5.53	2.89	TI	342.14	3.87
Dy	3.52	3.05	Ni	7.55	2.52	Tm	3.02	3.01
Eu	4.03	3.11	Np	9.56	3.05	U	11.07	3.02
Er	3.52	3.02	O	30.19	3.12	V	8.05	2.80
Es	6.04	2.94	Os	18.62	2.78	W	33.71	2.73
F	25.16	3.00	P	153.46	3.69	Xe	167.04	3.92
Fe	6.54	2.59	Pa	11.07	3.05	Y	36.23	2.98
Fm	6.04	2.93	Pb	333.59	3.83	Yb	114.72	2.99
Fr	25.16	4.37	Pd	24.15	2.58	Zn	62.39	2.46
Ga	208.81	3.90	Pm	4.53	3.16	Zr	34.72	2.78

Table S2. Lennard-Jones parameters and charges of adsorbates[2-5].

Atom	ε/k_B [K]	σ [Å]	Charge (e)
CH_4	151.16	3.7314	
H_2	24.692	3.0292	
N_N_2	36.0	3.31	-0.484
N_com	0	0	+0.964
O_O_2	49.0	3.02	+0.113
O_com	0	0	-0.226

Table S3. Kinetic diameter of CH_4, N_2, O_2 and H_2.

Gas	CH_4	N_2	O_2	H_2
Kinetic diameter (Å)	3.76	3.64	3.47	2.89

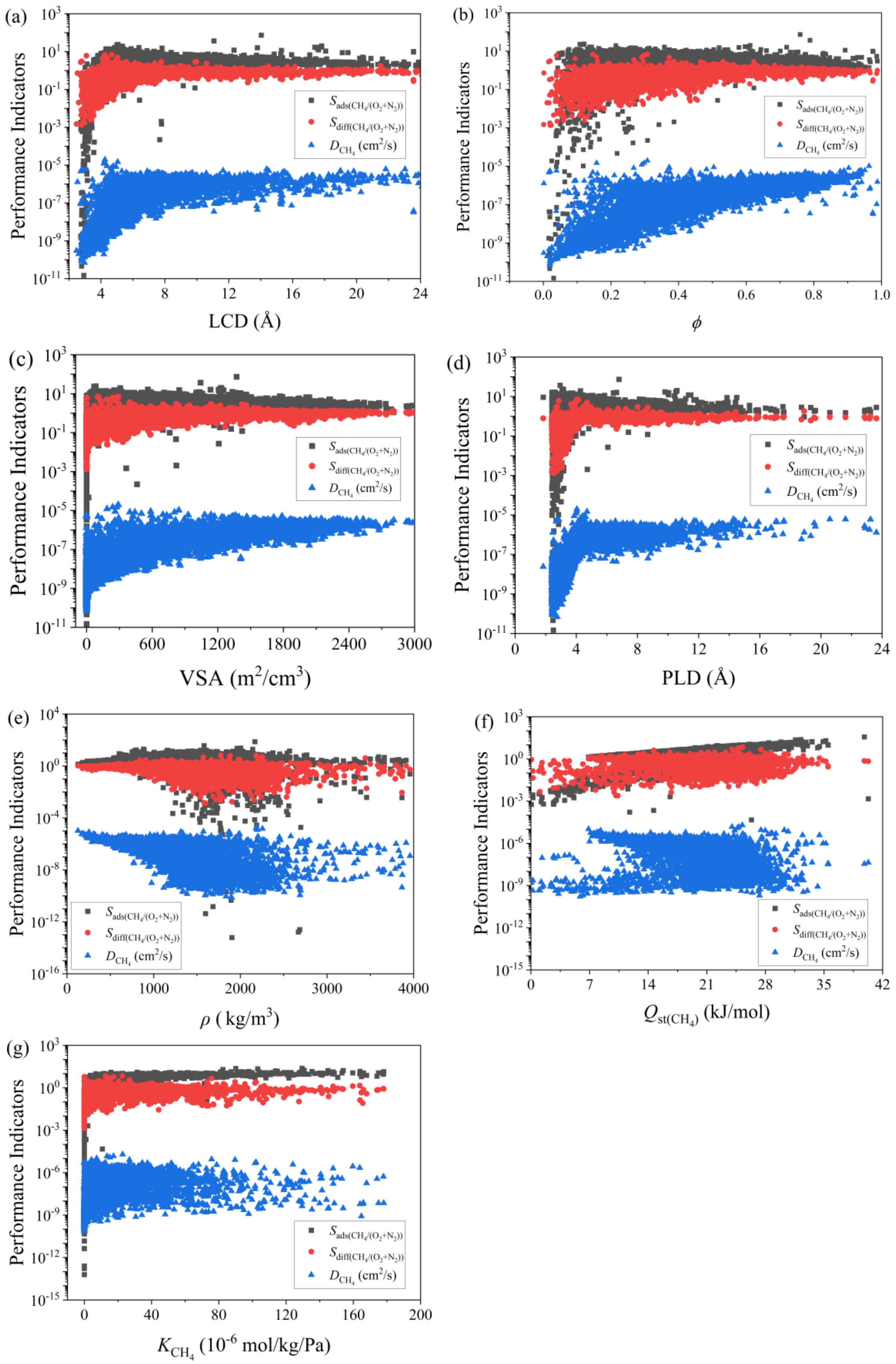

Figure S1. The relationship between seven descriptors (LCD, ϕ, VSA, PLD, ρ, $Q^0_{st(CH4)}$ and K_{CH_4}) of MOFs and three performance indexes (D_{CH_4}, $S_{ads(CH_4/O_2+N_2)}$ and $S_{diff(CH_4/O_2+N_2)}$).

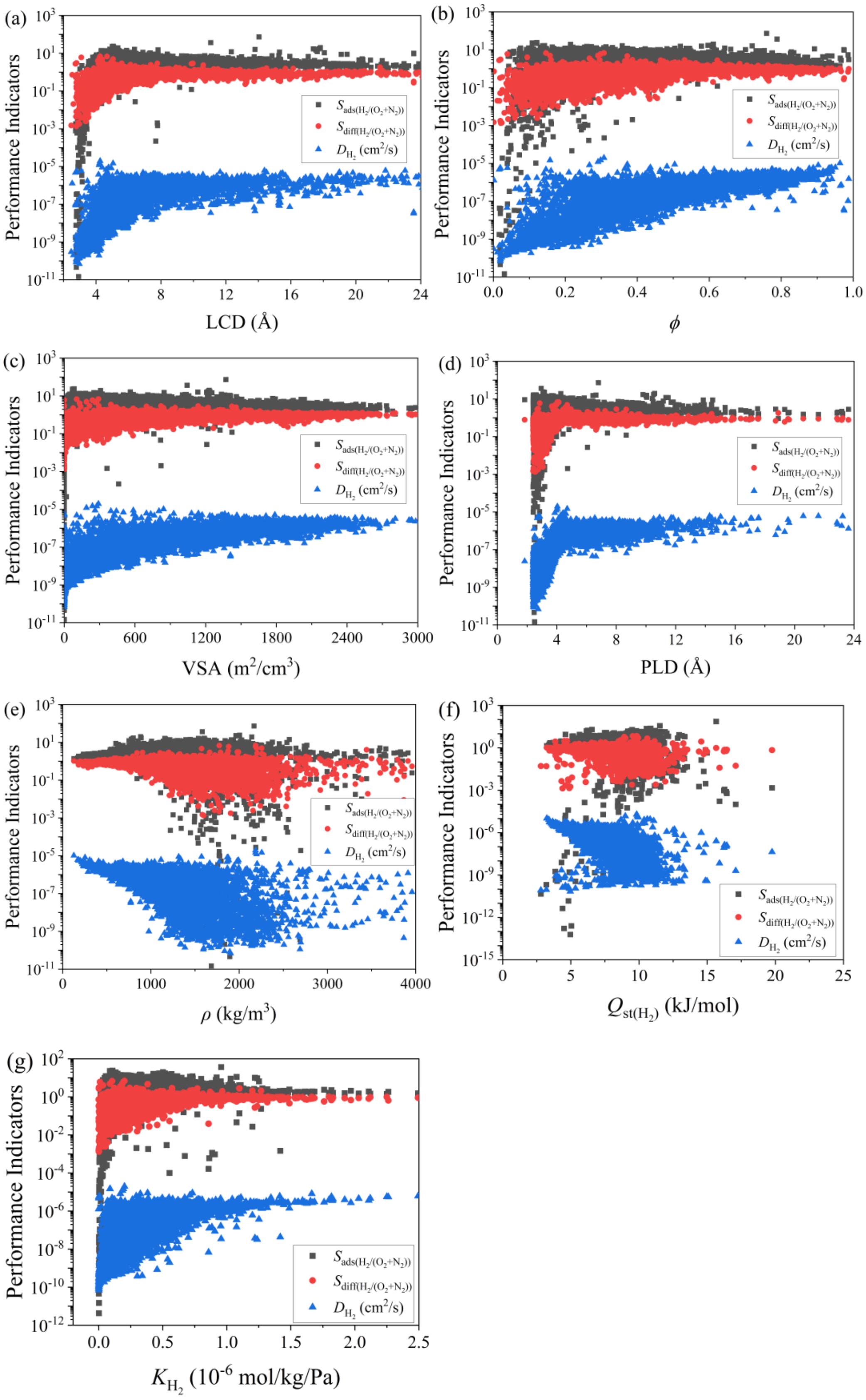

Figure S2. The relationship between seven descriptors (LCD, ϕ, VSA, PLD, ρ, $Q^0_{st(H2)}$ and K_{H_2}) of MOFs and three performance indexes (D_{H_2}, $S_{ads(H_2/O_2+N_2)}$ and $S_{diff(H_2/O_2+N_2)}$).

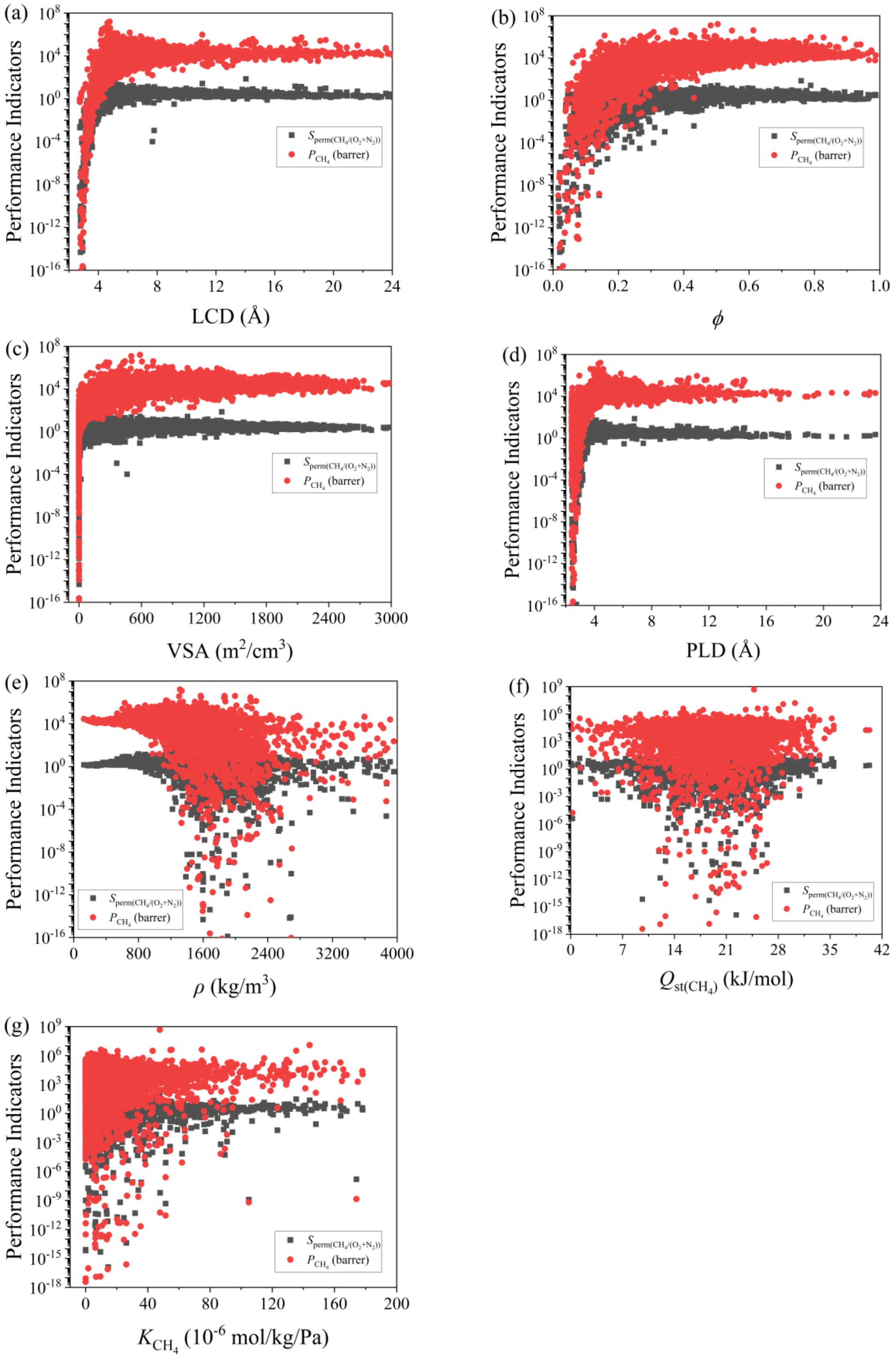

Figure S3. The relationship between seven descriptors (LCD, ϕ, VSA, PLD, ρ, $Q^0_{st(CH4)}$ and K_{CH_4}) of MOFMs and two performance indexes (P_{CH_4} and $S_{perm(CH_4/O_2+N_2)}$).

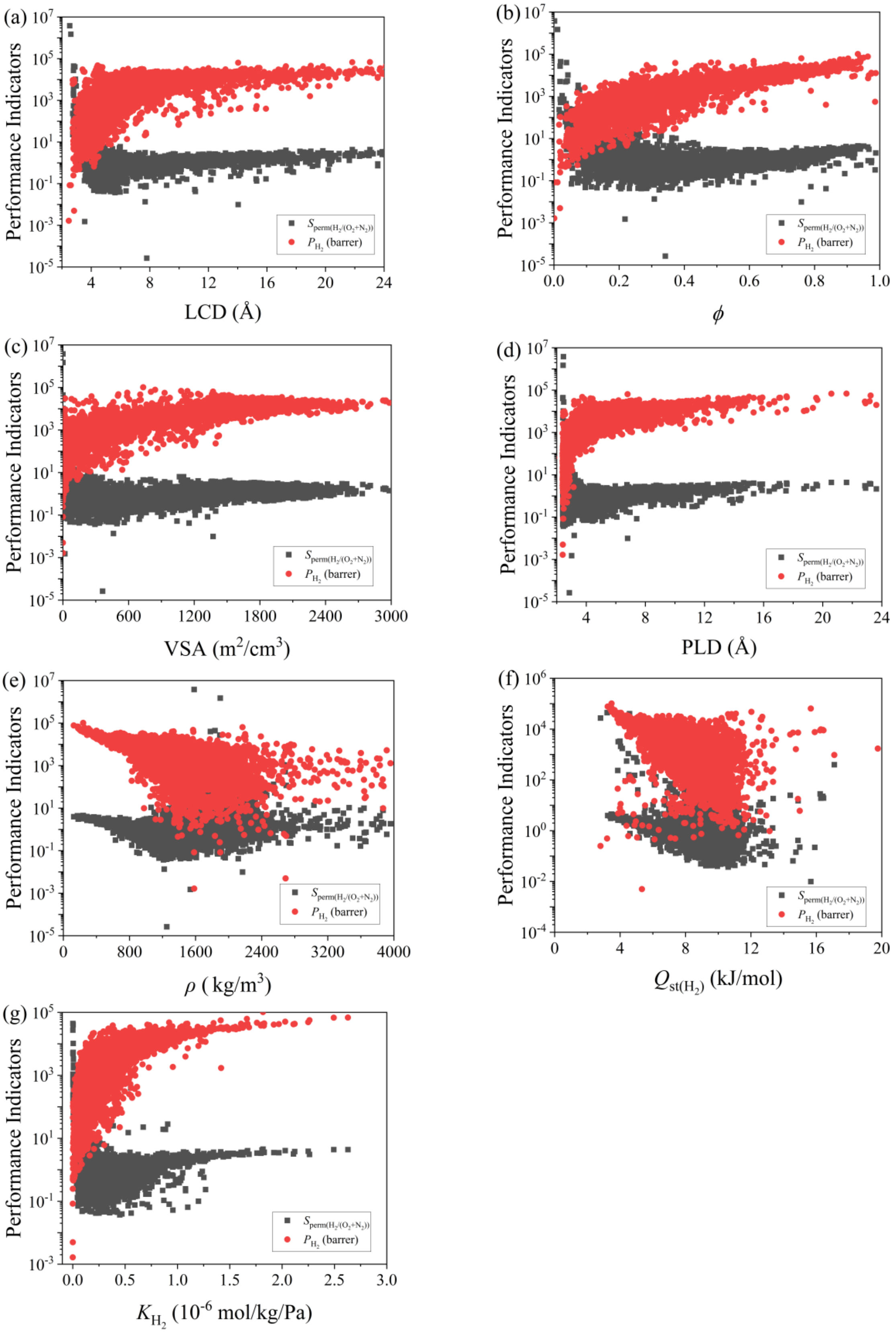

Figure S4. The relationship between seven descriptors (LCD, ϕ, VSA, PLD, ρ, $Q^0_{st(H2)}$ and K_{H_2}) of MOFMs and two performance indexes (P_{H_2} and $S_{perm(H_2/O_2+N_2)}$).

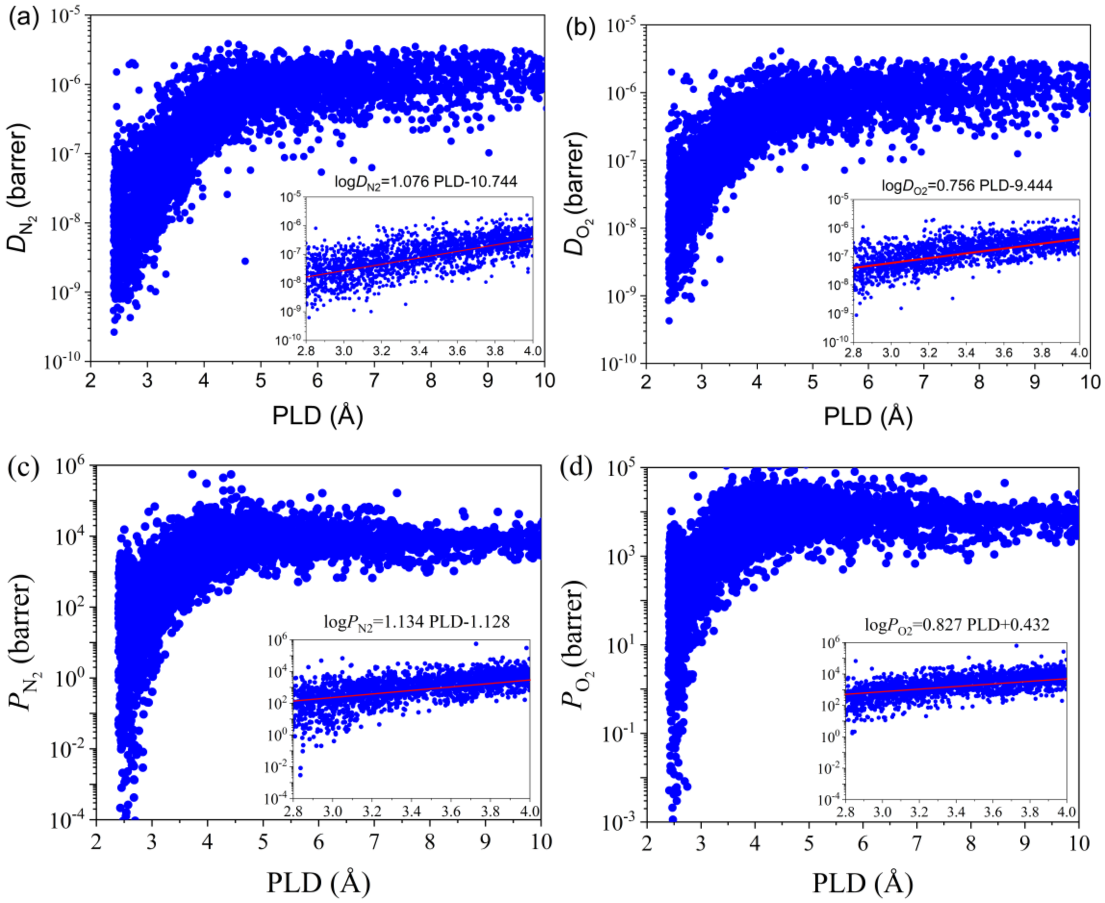

Figure S5. Diffusion coefficient D and Permeability P *versus* PLD for N_2 and O_2 in 6013 CORE-MOFs.

Machine learning methods

Tree-based pipeline optimization tool

Tree-based pipeline optimization tool (TPOT) is a great Python automated machine learning tool that uses genetic algorithms to programmatically optimize ML pipelines for automated feature selection and model selection. The tool enables automatic model generation to implement prediction tasks for classification or regression. TPOT will automate the most tedious parts of machine learning by intelligently exploring thousands of possible pipelines to find the one that best suits the target task. TPOT automates feature selection, feature preprocessing, feature construction, model selection, and parameter tuning. It's even possible to intelligently explore thousands of possible pipelines to automate the most tedious parts of machine learning to find the one that best fits the target task. Once the search is complete, it will also output a Python code pipeline that can run independently for subsequent optimization and learning. The model schematic is shown in Figure S6.

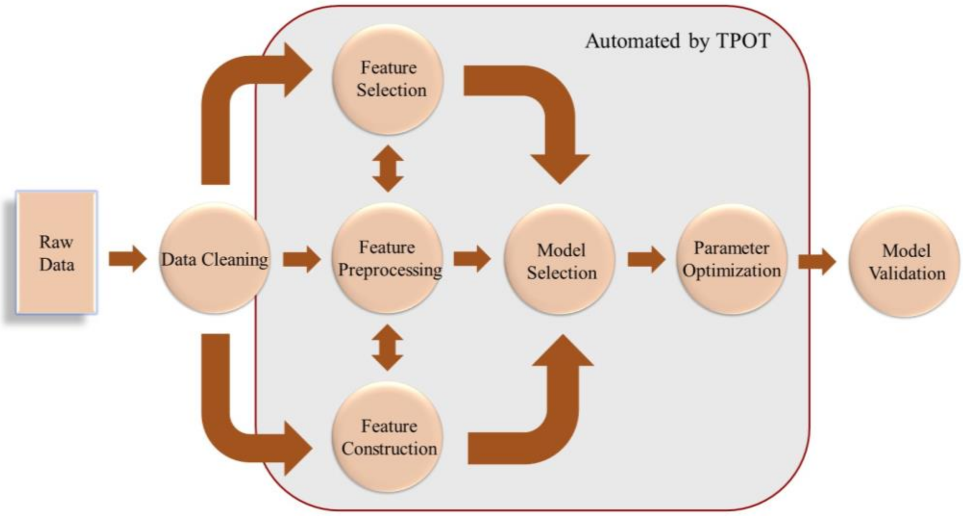

Figure S6. Tree-based pipeline optimization tool

Decision tree

Decision tree (DT) are a method of supervised learning that can be used for both classification and regression. On the DT nodes, eigenvalues are selected from the most important to the next important. The calculations are tried for each eigenvalue by a DT algorithm, and then the optimal classification is defined as the parent node after considering the attribution of each feature. Furthermore, the independent variable X_i is divided into two or more groups according to a certain splitting criterion. A tree is created through a plurality of splitting nodes. The most common DT algorithm is the binary branching principle, as shown in Figure S7. The independent variable X_i starts from the root node and is divided into two, and the DT is finally ended when it reaches the leaf nodes. The DT is created by an optimal splitting criterion. If the splitting criterion can make the dependent variable Y_i as a test set be the same as the calculated Y_i' in the leaf nodes, or within a specified error range, the calculated Y_i' are the output by DT.

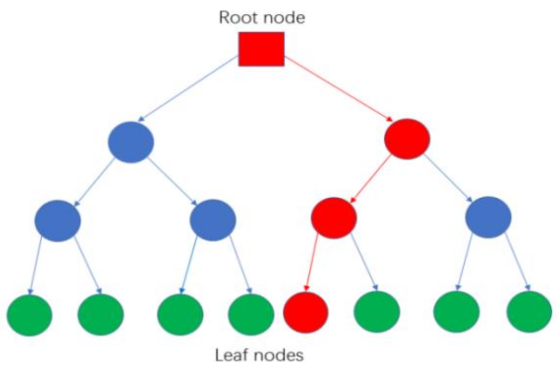

Figure S7. Decision tree.

Random forest

Random forest (RF) is also one of the most commonly used algorithms because it is simple and can be used for both classification and regression. It is an integrated algorithm based on the bagging approach, which refers to a model that uses multiple decision trees to train and predict samples. It contains multiple decision tree models, each of which grows well and continuously splits as input sample prediction. The new sample will be input to each decision tree with leaf nodes, and the final consequence is the mean value of each tree prediction result. The results of the whole model have high precision and generalization performance. Random forest is a flexible and easy-to-use machine learning algorithm that can get good results in most cases, even without hyperparameter tuning. We set up 200 trees in Figure S8.

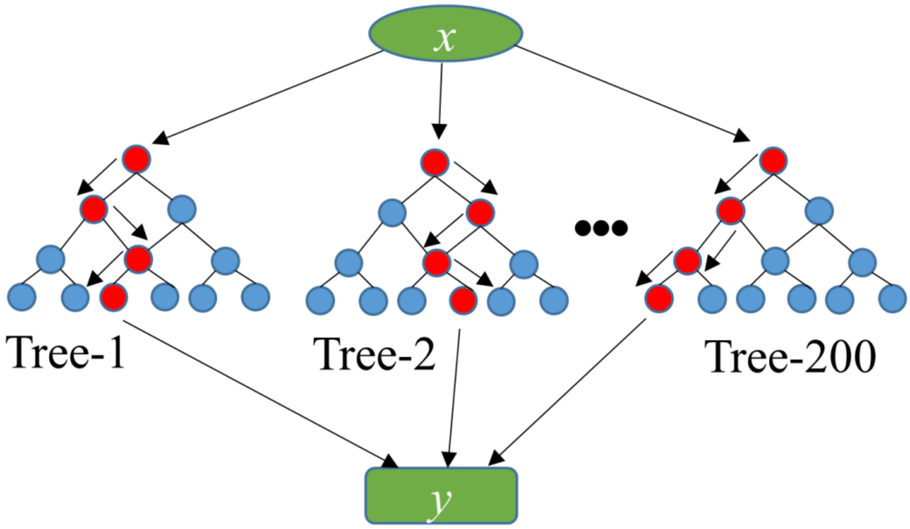

Figure S8. Random forest.

k-fold Cross Validation

In general, *k*-fold cross validation is used for model tuning to find the hyperparameters that makes the model generalization performance optimal. After the model is found, the model is retrained on all training sets, and the test set is used to make the final evaluation on the model performance, as shown in Figure S13. The so-called *k*-fold cross validation is to divide the data set into *k* parts (*k* =5) in equal proportion, namely, the training set (including the validation set) and the test set. One of them is used to validate the resulting model, and the *k* -1 of folded data are used to train the model. Finally, the parameter with the lowest average error is selected for each parameter.

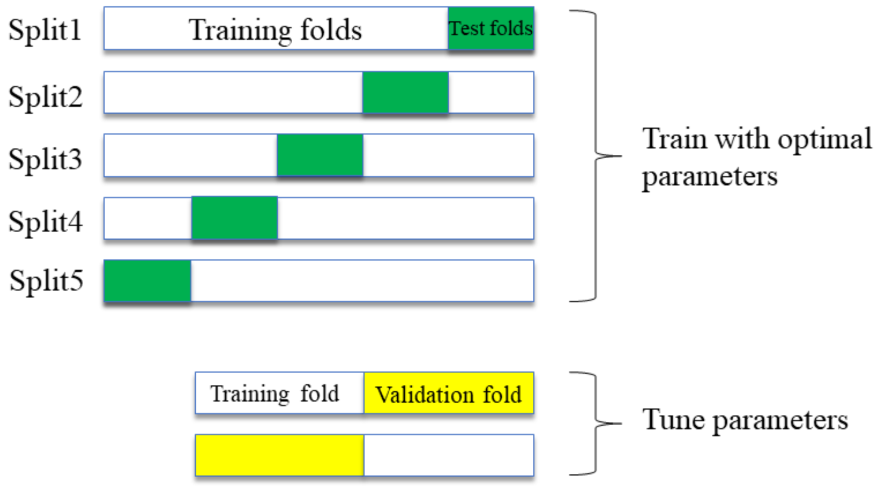

Figure S9. The diagram of *k*-fold cross-validation

Evaluation indicators of ML algorithms

Evaluation indicators can reflect the difference between the actual value and predicted value. Several evaluation indicators are widely used for regression models, e.g., mean absolute error (MAE), root-mean-square error (RMSE), and *R*, as calculated in equations S (1) - (3), where x_i is the GCMC simulated value, y_i is the value ML predicted, n is number of MOFs, and $\bar{y}$ is the average of the ML predicted values.

$$\text{MAE}=\frac{1}{n}\sum_{i=1}^{n}\left|x_i-y_i\right| \qquad \text{S (1)}$$

$$\text{RMSE}=\frac{1}{n}\sum_{i=1}^{n}(x_i-y_i)^2 \qquad \text{S (2)}$$

$$R=\sqrt{1-\frac{\sum_{i=1}^{n}(xi-yi)^2}{\sum_{i=1}^{n}(yi-\bar{y})^2}} \qquad \text{S (3)}$$

Table S4. The constraints for screening the best MOFs/MOFMs under each system.

Applications		CH_4/O_2+N_2	H_2/O_2+N_2
MOFs	D (cm^2/s)	10^{-6}	10^{-6}
	S_{ads}	5	5
	S_{diff}	4	10
MOFMs	P (barrer)	3000000	5000
	S_{perm}	20	16

Table S5. Design Strategies of MOFs and MOFMs with high performance

No.	CSD	Design types				The percentage of $S_{ads(CH_4/O_2+N_2)}$ increased	The percentage of $S_{diff(CH_4/O_2+N_2)}$ increased	The percentage of D_{CH_4} increased	The percentage of P_{CH_4} increased	The percentage of $S_{perm(CH_4/O_2+N_2)}$ increased
			Metal	Linker	Topology					
1	PARHEW[a]	Topology	Cd	$C_9H_6O_6$	rtl 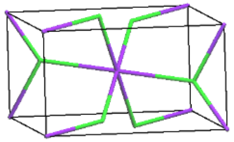	55.43%	42.12%	79.39%	57.37%	120.91%

	MITGUR01[b]			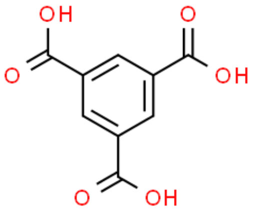	mmm 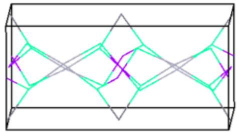					
2	VEJYIT[a]		Zn	$C_3H_4N_2$ 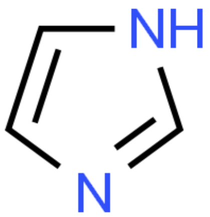	crb 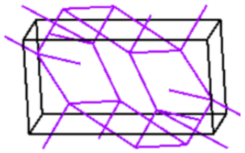	44.18%	12.64%	516.52%	1789.10%	62.68%
	IMIDZB10[b]				sod 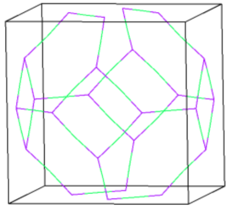					

3	cg300979c_si_002[a]		Mn	$C_9H_6O_6$ 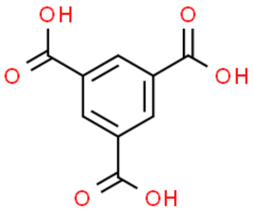	pyr 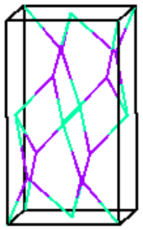	-10.52%	23.79%	262.88%	454.09%	9.20%
	DAPBIH[b]				tbo 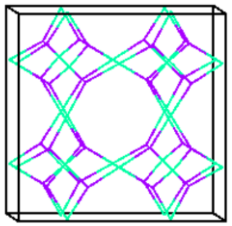					
4	CAXZOS[a]		Co	$C_{12}H_9NO_2$	dia 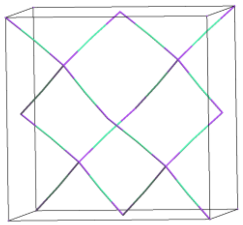	30.53%	91.72%	217.40%	241.14%	156.68%

	ZILBED[b]			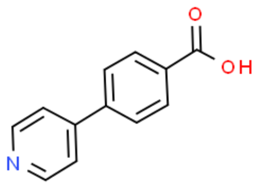	pts 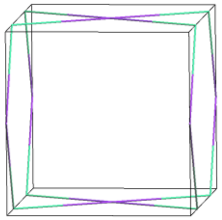					
5	OSAVEK[a]	Linker	Ca	$C_8H_6O_6$ 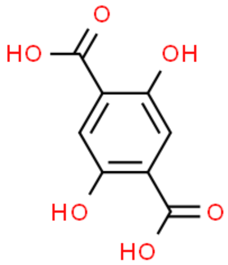	dmd 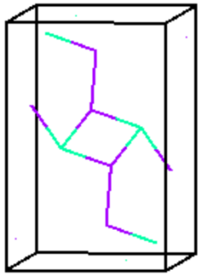	16.50%	22.17%	58.20%	238.08%	43.75%
	PARFOF[b]			$C_6H_{10}O_4$ 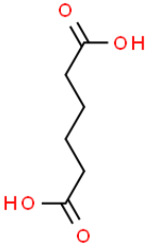						

6	BEDHOJ[a]		Ni	$C_8H_6N_2O_2$ 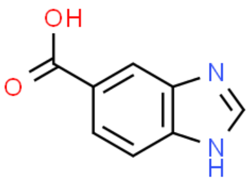	dia 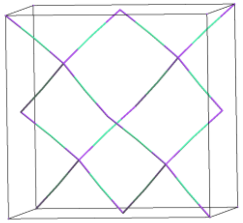	38.11%	175.37%	3234.62%	84.37%	281.02%
	UFATEA01[b]			$C_6H_5NO_2$ 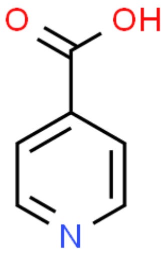						
7	ZISYAD[a]		Cu	$C_{45}H_{32}N_4$ 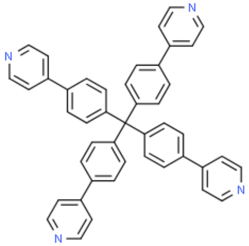	dia 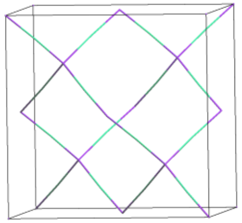	5.28%	43.84%	122.42%	244.48%	50.92%

	ja5069855_si_002[b]			$C_9H_7N_3O_2$ 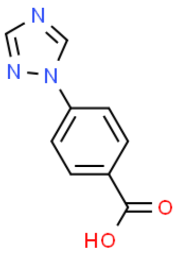						
8	PEPLIG[a]<br>jp102463p_si_002[b]	Metal	V<br>Cr	$C_8H_6O_4$ 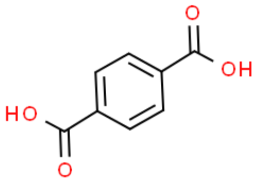	rna 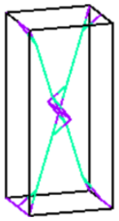	42.83%	44.22%	61.39%	293.00%	107.93%

9	SACDOR[a]		Zn	$C_{18}H_{12}N_6$ 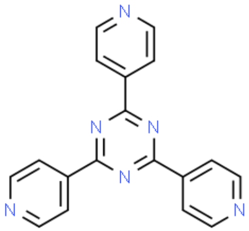	srs 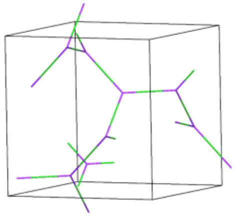	-5.89%	85.28%	128.79%	167.43%	77.63%
	SETPEO[b]		In							
10	FOHCIP[a]		Cd,Hg	SCNH 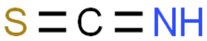	mmt 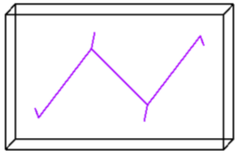	4.64%	27.64%	26.63%	52.93%	33.95%

	XADGAM[b]		Mn,Hg							
No.	CSD	Design types				The percentage of $S_{ads(H_2/O_2+N_2)}$ increased	The percentage of $S_{diff(H_2/O_2+N_2)}$increased	The percentage of D_{H_2} increased	The percentage of P_{H_2} increased	The percentage of $S_{perm(H_2/O_2+N_2)}$ increased
			Metal	Linker	Topology					
11	GUPBUP[a]	Topology	Cd	$C_4H_6N_2$ 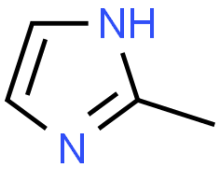	sod 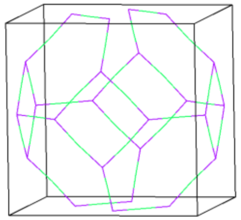	182.78%	-7.03%	170.08%	503.81%	166.30%

	GUPBOJ01[b]				ict 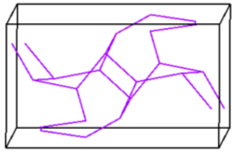					
12	XAZGEK[a]		Ag	$C_6H_{12}N_4$ 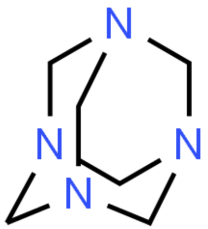	mog 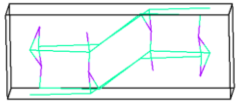	99.48%	143.40%	490.68%	657.25%	378.40%
	BAHMAZ[b]				hcb 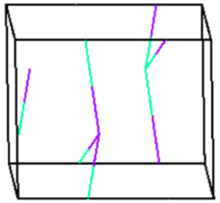					

13	XIGHIF[a]		Zn	$C_{18}H_{12}N_6$ 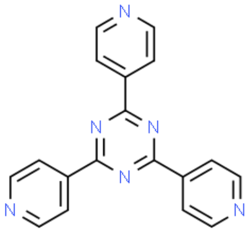	ths 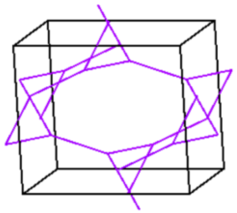	28.32%	6.26%	85.94%	53.93%	36.29%
	SOMCON[b]				srs 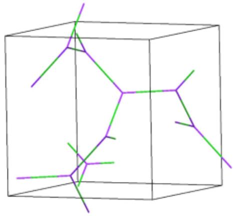					
14	PARHAS[a]	Linker	Ca	$C_6H_{10}O_4$ 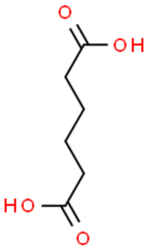	dmd 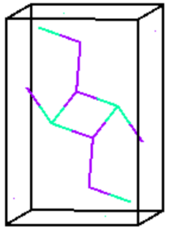	-44.12%	175.99%	446.30%	1683.03%	30.60%

	LEZZEX[b]			$C_8H_{12}O_4$ 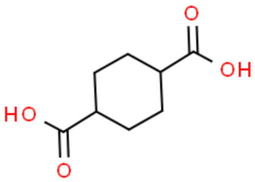						
15	QOZSIJ[a]		Zn	$C_{35}H_{23}N_3O_4$ 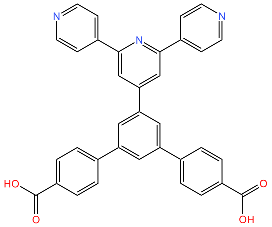	ths 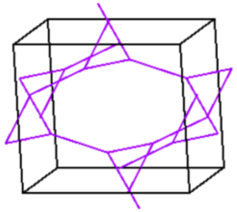	47.38%	7.51%	173.53%	301.08%	60.19%
	LUCDOE[b]			$C_{18}H_{12}N_6$ 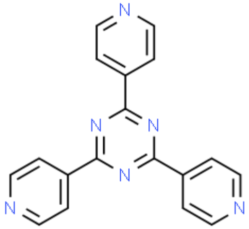						

16	FUSWIA[a]		Cu	$C_6H_4N_5$ 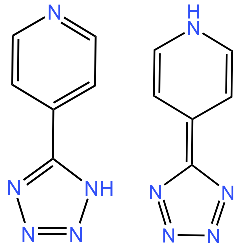	bcu 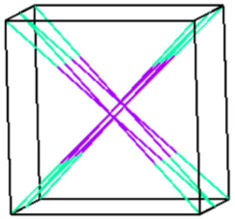	297.28%	21.24%	73.36%	34.53%	382.40%
	HAWZEM[b]			$C_6H_5O_2N$ 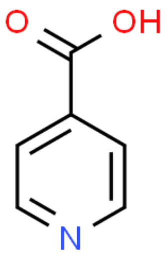						
17	PIBPIA[a]	Metal	Zn	$C_8H_6O_4$	bcg	63.47%	27.63%	71.57%	114.93%	106.77%

	VEGMIE01[b]		Co	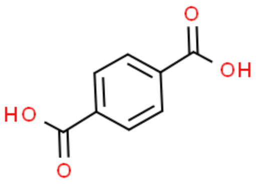	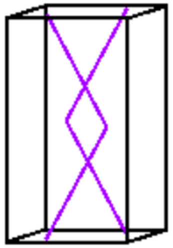					
18	FUTCAZ[a]		Mn	$C_9H_6O_6$ 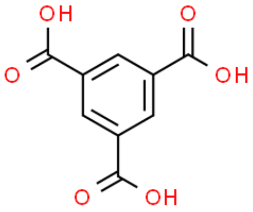	srs 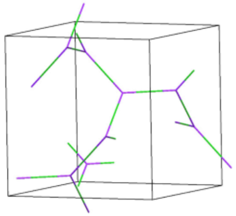	160.71%	37.18%	136.66%	207.41%	245.74%
	HUYJUG[b]		Ni							

19	MUVJIX[a]		Fe	$C_9H_6N_{12}$ 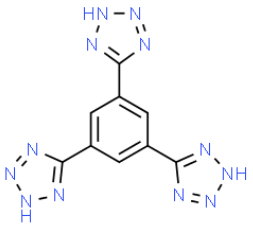	the 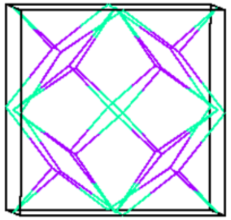	14.78%	26.19%	182.26%	197.29%	44.29%
	VEXYON[b]		Cu							
20	HEBTEP[a]		Zn	$C_{12}H_8O_4$	lim 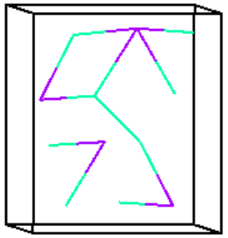	67.05%	102.80%	952.58%	644.99%	223.63%

	SETFUT[b]		Cd	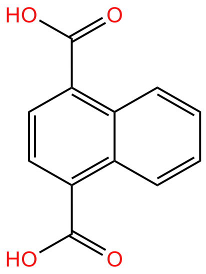						

[a] High-performance MOFs in the same group; [b] Poor performance MOFs in the same group.

References

1 . A. K. Rappé; C. J. Casewit; K. S. Colwell; W. A. Goddard III; Skif., W. M. Uff a full periodic table force field for molecular mechanics and molecular dynamics simulations. *J. Am. Chem. Soc.* **1992**, *114*, 10024-10035.

2 . Potoff, J. J.; Siepmann, J. I. Vapor-liquid equilibria of mixtures containing alkanes, carbon dioxide, and nitrogen. *AIChE J.* **2001**, *47*, 1676-1682.

3 . Stoll, J.; Vrabec, J.; Hasse, H. Vapor-liquid equilibria of mixtures containing nitrogen, oxygen, carbon dioxide, and ethane. *AIChE J.* **2003**, *49*, 2187-2198.

4 . Martin MG, S. J. Transferable Potentials for Phase Equilibria. 1. United-Atom Description of n-Alkanes. *J. Phys. Chem. B* **1998**, *102*, 2569-2577.

5 . Shah, M. S.; Tsapatsis, M.; Siepmann, J. I. Development of the Transferable Potentials for Phase Equilibria Model for Hydrogen Sulfide. *J. Phys. Chem. B* **2015**, *119*, 7041-7052.
